# A bird’s eye view on the use of whole exome sequencing in rare congenital ophthalmic diseases

**DOI:** 10.1038/s10038-024-01237-6

**Published:** 2024-03-08

**Authors:** Jessica Zucco, Federica Baldan, Lorenzo Allegri, Elisa Bregant, Nadia Passon, Alessandra Franzoni, Angela Valentina D’Elia, Flavio Faletra, Giuseppe Damante, Catia Mio

**Affiliations:** 1 Institute of Medical Genetics, Azienda Sanitaria Universitaria Friuli Centrale (ASUFC), Udine, Italy; 2 Department of Medicine (DMED), University of Udine, Udine, Italy

**Keywords:** Genetics research, Next-generation sequencing

## Abstract

Phenotypic and genotypic heterogeneity in congenital ocular diseases, especially in anterior segment dysgenesis (ASD), have created challenges for proper diagnosis and classification of diseases. Over the last decade, genomic research has indeed boosted our understanding in the molecular basis of ASD and genes associated with both autosomal dominant and recessive patterns of inheritance have been described with a wide range of expressivity. Here we describe the molecular characterization of a cohort of 162 patients displaying isolated or syndromic congenital ocular dysgenesis. Samples were analyzed with diverse techniques, such as direct sequencing, multiplex ligation-dependent probe amplification, and whole exome sequencing (WES), over 20 years. Our data reiterate the notion that *PAX6* alterations are primarily associated with ASD, mostly aniridia, since the majority of the cohort (66.7%) has a pathogenic or likely pathogenic variant in the *PAX6* locus. Unexpectedly, a high fraction of positive samples (20.3%) displayed deletions involving the 11p13 locus, either partially/totally involving *PAX6* coding region or abolishing its critical regulatory region, underlying its significance. Most importantly, the use of WES has allowed us to both assess variants in known ASD genes (i.e., *CYP1B1*, *ITPR1*, *MAB21L1*, *PXDN*, and *PITX2*) and to identify rarer phenotypes (i.e., MIDAS, oculogastrointestinal-neurodevelopmental syndrome and Jacobsen syndrome). Our data clearly suggest that WES allows expanding the analytical portfolio of ocular dysgenesis, both isolated and syndromic, and that is pivotal for the differential diagnosis of those conditions in which there may be phenotypic overlaps and in general in ASD.

## Introduction

Congenital ocular malformations may affect any part of the eye and the ocular adnexa. The major early morphological events in the development of the eye can be broadly summarized into four main steps: (i) formation of the optic vesicle, (ii) induction of lens, (iii) organization of early retina, and (iv) fusion of the optic fissure [1]. Developmental defects may occur in isolation or as part of a larger systemic malformation syndrome [2]. A significant number of congenital ocular anomalies indeed involve the anterior segment of the eye.

Congenital anterior segment dysgenesis (ASD) refers to a spectrum of congenital disorders involving abnormal development of the anterior segment of the eye, i.e., the cornea, iris, trabecular meshwork, ciliary body, and lens [3]. Approximately 50% of ASD patients will develop glaucoma, typically at a young age [4]. ASD encompasses a variety of conditions, such as Axenfeld–Rieger syndrome (ARS), aniridia, coloboma, congenital glaucoma, Gillespie syndrome, microphthalmia, and Peters anomaly (PA), among others. The main characteristics of these disorders are summarized in Supplementary Table 1.

The discrete range of clinical manifestations and the common overlapping characteristics of the aforementioned diseases make their classification challenging. In addition, several genes contribute to multiple phenotypes, adding to the complexity of the phenotype–genotype correlations and genetic diagnostic accuracy [5].

Over the last decade, genomic research has indeed boosted our understanding in the molecular basis of ASD. Identification of the genetic changes underlying ASD has gradually led to recognizing that some of these conditions may be parts of a disease spectrum [6]. Whether *PAX6* haploinsufficiency (caused by heterozygous loss of function variants in the *PAX6* gene or its associated regulatory regions spanning the 11p13 locus) is able to explain about 90–98% of all aniridia cases, this is not true for some of the other conditions included in ASD. For example, the 13q14 locus (RIEG2) has been strongly associated to ARS but the altered gene causative of this disease has not been yet identified [7]. Although mutations of *CYP1B1*, *LTBP2*, *MYOC*, *FOXC1*, *TEK*, and *ANGPT1* have been implicated in primary congenital glaucoma (PCG), the inner molecular bases of the disease remain largely undisclosed [8], leading to a detection rate of 10–40% that mostly varies across ethnic groups [9]. Lastly, despite the number of genes known to play a role in PA, a genetic diagnosis is found in about 25% of cases [10].

Notwithstanding the implementation of diagnostic techniques, the pathomechanisms of some of these conditions are far from being elucidated.

In this scenario, the advent of high-throughput DNA sequencing technologies has led to the identification of many genes and DNA sequence variants implicated in human eye disorders and contributed to the progress in understanding the processes driving the development of the eye [11]. Nonetheless, there is a lack of systematic investigation into the diagnostic utility of next-generation sequencing (NGS) in the entire group of ASD patients. To date, most studies focus on a particular phenotypic subset or gene set, and such studies suggest there may be a detection rate of <10–40% in the broader cohort [5].

Given these premises, here we describe the molecular characterization of cohort of 162 patients displaying isolated or syndromic congenital ocular dysgenesis.

## Materials and methods

### Patient and sample collection

This study uses clinical information and biological samples from 162 individuals referred to the Institute of Medical Genetics of the Azienda Sanitaria Universitaria Friuli Centrale (ASUFC) of Udine (Italy), from 2003 to 2023. Written informed consent for research was obtained from all patients for use of their samples in genetic studies. This study was approved by Institutional Review Board (IRB-DAME, Prot IRB: 191/2023).

Samples were analyzed for intragenic *PAX6* alterations by direct sequencing and multiplex ligation-dependent probe amplification (MLPA) until 2019 and whole exome sequencing (WES) from 2019 to 2023. Samples tested negative prior 2019 were reanalyzed by WES. Copy number variants were confirmed by microarray comparative genomic hybridization (aCGH).

### DNA extraction

Genomic DNA was extracted from peripheral blood samples collected into 10 ml EDTA K2 blood collection tubes using the QIAsymphony® SP/AS instrument (Qiagen, Hilden, Germany) according to the manufacturer’s instruction. DNA quantity was estimated using the Qubit™ dsDNA HS Assay Kit on a Qubit 4.0 Fluorometer (Thermo Fisher Scientific, Waltham, MA, USA).

### Sanger sequencing

Sanger sequencing of *PAX6* exons 4–13 and of the 15 *PAX6* enhancers was performed as previously described [12–14]. Amplification was performed using 150 ng of DNA and GoTaq® Colorless Master Mix (Promega, Madison, WI, USA). PCR primer sequences are available on demand. The amplified products were analyzed by direct sequencing using the Big Dye Terminator Cycle Sequencing Kit v3.1 and capillary electrophoresis on the 3500 Dx Series Genetic Analyzer (Applied Biosystems, Waltham, MA, USA).

### Whole exome sequencing and data analysis

Barcoded libraries were generated from 50 ng of DNA per sample (*n* = 64). The exonic regions and flanking splice junctions (±25 bp flanking each exon) of about 22,000 coding genes were captured using the WholEX pro sequencing kit (4bases SA, Manno, Switzerland). Sequencing was performed in paired-end 2 × 150 bp on a NextSeq system (Illumina Inc., San Diego, CA, USA).

Reads were aligned to human genome build GRCh38/hg38 and variant calling were performed with the Varsome Clinical platform (Saphetor SA, Lausanne, Switzerland). Variant annotation and classification were performed with eVai (enGenome, Pavia, Italy). A minimum depth coverage of 20× and a minimum alternate allele frequency of 20% (VAF ≥ 20%) were considered suitable for analysis. Variants with frequency <0.1% in population-based databases (i.e., gnomAD), exonic missense, splicing, stop-gain, stop-loss, and frameshift insertion and deletion variants were retained for further evaluation. The following public databases were used for the interpretation of the variants: HGMD Professional (https://my.qiagendigitalinsights.com/bbp), LOVD (https://databases.lovd.nl/shared/genes), ClinVar (https://www.ncbi.nlm.nih.gov/clinvar/), Varsome Premium (https://varsome.com/).

Variants were classified according to the American College of Medical Genetics and Genomics (ACMG) guidelines [15]. Candidate variants were classified as potentially disease-causing based on ACMG criteria, reports of previous cases with a comparable phenotype, animal models, and/or functional studies.

### Multiplex ligation-dependent probe amplification

The SALSA MLPA Probemix P219 PAX6 (MRC-Holland, Amsterdam, The Netherlands) was used to detect deletions or duplications in the 11p13-14 region, which includes the *PAX6* and *WT1* genes, and the *SOX2* gene on 3q26, which are associated with hereditary ocular malformations. All procedures and data analysis were performed as indicated by the manufacturer. Capillary electrophoresis was performed on a 3500 Dx Series Genetic Analyzer (Applied Biosystems) using the LIZ 500 Size Standard v2.0. Data were analyzed using Coffalyser.Net™ Software (MRC-Holland) following manufacturer’s instructions.

### Microarray-based comparative genomic hybridization (aCGH)

Array CGH analyses were performed using the Agilent Human Genome CGH oligonucleotide array 180 K following the manufacturer’s instructions (Agilent Technologies, Santa Clara, CA, USA). Images were analyzed with the Agilent Feature Extraction, Genomic Workbench 6.5.018 Lite Edition Software, and genomic coordinates were evaluated according to GRCh37/hg19. Genes located in the deleted area were investigated by the UCSC genome browser database (http://genome.ucsc.edu, hg19).

## Results

### Cohort characteristics

**Fig. 1:**
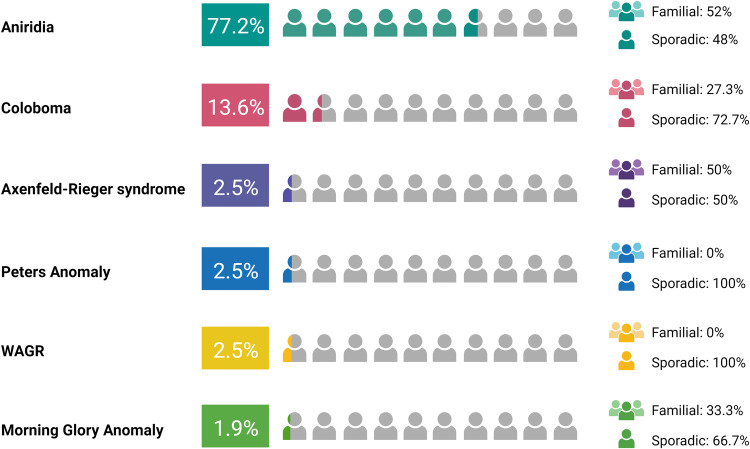
Summary of patients’ phenotypes. The figure represents the percentage of patients with a diagnosis of aniridia, coloboma, Axenfeld–Rieger syndrome, Peters anomaly, WAGR, and morning glory anomaly, together with the percentage distribution between familial and sporadic cases

Our cohort include 162 patients referred to the Institute of Medical Genetics at the ASUFC between 2003 and 2023 for genetic testing for ASD. Most of the cohort displayed isolated aniridia (*N* = 125), the remaining ones presented coloboma (*N* = 22), Axenfeld–Rieger syndrome (*N* = 4), Peters anomaly (*N* = 4), WAGR (*N* = 4), and morning glory anomaly (*N* = 3) (Fig. 1). The majority of patients exhibited sporadic disease (54.3%).

### Frequency of 11p13 alterations

**Fig. 2:**
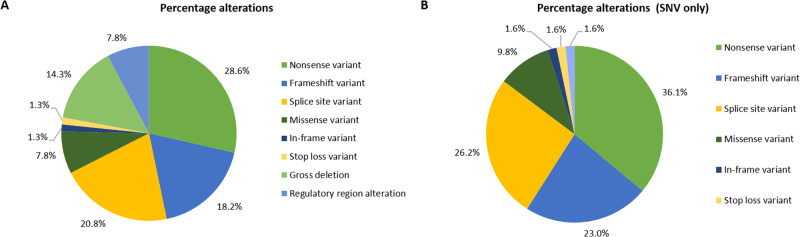
Distribution of PAX6 alterations in our cohort. **A** Percentage of alterations affecting the 11p13 locus considering both SNVs and SVs. **B** Percentage of SNVs affecting the 11p13 locus found in our cohort

In the first analytical setting only *PAX6* gene alterations were evaluated. SNVs and SVs were assessed by direct sequencing and MLPA, respectively. Moreover, considering that *PAX6* is surrounded by ultra-conserved cis-regulatory sequences (CREs), direct sequencing of its 15 enhancer sequences was performed [16, 17]. Overall, 66.7% of samples bore a pathogenic or likely pathogenic aberration in the *PAX6* locus (*N* = 108), either affecting *PAX6* coding region or its regulatory domains. Merging familial cases, gross deletions were found in 20.8% (16 out of 77) of patients diagnosed with isolated or syndromic aniridia. A total of 31.25% (5 out of 16) of these deletions exclusively involve *PAX6* CREs embedded within *DCDC1*, *DNAJC24*, *ELP4*, and *IMMP1L* genes, located downstream *PAX6*. Figure 2 depicts variant distribution in the *PAX6* locus.

**Fig. 3:**
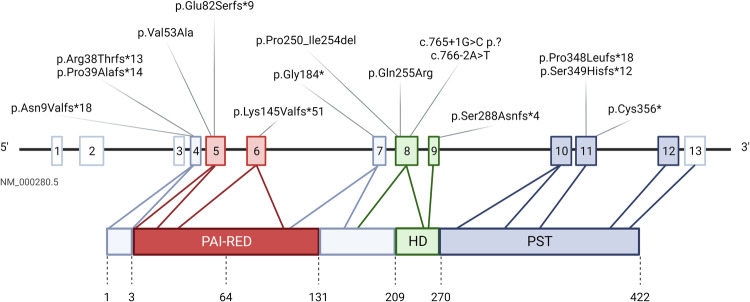
Spectrum of novel coding PAX6 mutations assessed in this study. The figure represents novel variants, i.e., alterations not included in publicly available databases, within the *PAX6* coding region identified in our cohort. Boxes represent the 13 PAX6 exons and their colors represent respective protein domains. Moreover, canonical PAX6 protein is represented with its functional domains. PAI-RED paired domain; HD homeodomain; PST proline–serine–threonine domain

A total of 61 pathogenic or likely pathogenic variants were detected in the *PAX6* gene from 19 pedigrees and additional 42 sporadic patients, including 15 novel and 35 previously reported variants (i.e., included in ClinVar and/or HGMD databases). Considering unique SNVs (*N* = 50), 52% of variants falls within the known *PAX6* mutation hotspots, i.e., located in known methylated CpG islands between exons 7–12 [18]. A total of 20% of SNVs are located in *PAX6* exon 5, which turned out to be the most affected exon both in our cohort and those formerly reported in the literature. Besides, one patient displayed a non-coding SNVs located in the ultra-conserved PAX6 enhancer, whose pathogenicity was already discussed by Bhatia et al. [19]. Table 1 summarizes pathogenic or likely pathogenic findings within the *PAX6* locus. Figure 3 depicts the location of novel *PAX6* variants assessed in our cohort.

**Table 1:** *PAX6* pathogenic or likely pathogenic alterations found in our cohort

ID	Disease	Inheritance	Gene	Variant	Genomic position (hg19)
A1	Aniridia	Sporadic	PAX6	c.357C>G p.Ser119Arg	chr11-31823109-G-C
A3	Aniridia	Sporadic	PAX6	c.607C>T p.Arg203*	chr11-31816253-G-A
A6	Aniridia	Sporadic	PAX6	c.607C>T p.Arg203*	chr11-31816253-G-A
A13	Aniridia	Sporadic	PAX6	c.916+1G>A p.Arg203*	chr11-31815199-C-T
A18 A19 A29	Aniridia	Familial	DCDC1, DNAJC24, ELP4, IMMP1L	-^d^	arr[GRCh37]11p13(30953737_31685847)x1
A20	Aniridia	Sporadic	PAX6	c.357C>G p.Ser119Arg	chr11-31823109-G-C
A23	Aniridia	Sporadic	PAX6	c.771G>A^c^ p.Trp257*	chr11-31815345-C-T
A28	Aniridia	Sporadic	ARL14EP, DCDC1, DNAJC24, ELP4, FSHB, IMMP1L, MPPED2, PAX6, RCN1	-	arr[GRCh37]11p13p14.1(30255684_31907122)x1
A30	Aniridia	Sporadic	PAX6	c.357+1G>A p.?	chr11-31823108-C-T
A35	Aniridia	Sporadic	PAX6	c.110_117del p.Ala37Valfs*16	chr11-31824276-CGGCCGGG-
A40 A85	Aniridia	Sporadic	PAX6	c.357+1G>A p.?	chr11-31823108-C-T
A43 A55 A56 A57 A59	Aniridia	Familial	DCDC1, DNAJC24, ELP4, IMMP1L	-^d^	arr[GRCh37]11p13(31030697_31755156)x1
A48 A49	Aniridia	Familial	PAX6	c.375_376dup p.Val126Glufs*22	chr11-31822386--CT
A51	Aniridia	Sporadic	PAX6	c.406C>T p.Gln136*	chr11-31822356-G-A
A54	Aniridia	Sporadic	PAX6	c.244_245del^a^ p.Glu82Serfs*9	chr11-31823221TC--
A61	Aniridia	Sporadic	PAX6	c.607C>T p.Arg203*	chr11-31816253-G-A
A63 A64	Aniridia	Sporadic	DCDC1, DNAJC24, ELP4, IMMP1L	-^d^	arr[GRCh37]11p13(31212097_31751004)x1
A67	Aniridia	Sporadic	PAX6	c.3G>A p.Met1Ile	chr11-31827957-C-T
A69	Aniridia	Familial	DCDC1, DNAJC24, ELP4, IMMP1L, PAX6, RCN1	-^d^	arr[GRCh37]11p13(31327164_31876477)x1
A70	Aniridia	Familial	PAX6	c.781C>T p.Arg261*	chr11-31815335-G-A
A71A A71B	Aniridia	Familial	PAX6	c.358-1G>C p.?	chr11-31822405-C-G
A72	Aniridia	Sporadic	PAX6	c.180T>A p.Tyr60*	chr11-31823286-A-T
A73	WAGR	Sporadic	CCDC73, CSTF3, DCDC1, DEPDC7, DNAJC24, EIF3M, ELP4, IMMP1L, PAX6, PRRG4, QSER1, RCN1, TCP11L1, WT1	-^d^	arr[GRCh37]11p14.1p13(30638320_33182162)x1
A74 A75	Aniridia	Familial	PAX6	c.607C>T p.Arg203*	chr11-31816253-G-A
A81A A81B A81C A81D	Aniridia	Familial	DCDC1, DNAJC24, ELP4, IMMP1L, PAX6	-	arr[GRCh37]11p13(31329311_31828397)x1
A82	Aniridia	Sporadic	DCDC1, DNAJC24, ELP4, IMMP1L	-^d^	arr[GRCh37]11p14.1p13(30902664_31802443)x1
A83	Aniridia	Sporadic	PAX6	c.520C>T p.Gln174*	chr11-31822242-G-A
A86A A86B A86C A86D A86E A86F	Aniridia	Familial	PAX6	-	arr[GRCh37]11p13(31827945_31828010)x1
A87	Aniridia	Sporadic	PAX6	c.718C>T p.Arg240*	chr11-31815627-G-A
A88	Aniridia	Sporadic	PAX6	-	arr[GRCh37]11p13(31816347_31827834)x1
A89	Aniridia	Familial	PAX6	c.718C>T p.Arg240*	chr11-31815627-G-A
A91	Aniridia	Sporadic	PAX6	c.818dup p.Asn273Lysfs*11	chr11-31815298--T
A92	Aniridia	Familial	PAX6	c.1041_1053del^a^ p.Ser349Hisfs*12	chr11-31812388-GGTCTGGCTGGGG-
A95	Aniridia	Sporadic	PAX6	c.357+1G>A p.?	chr11-31823108-C-T
A96	Aniridia	Familial	PAX6	c.141+4A>G p.?	chr11-31824248-T-C
A110	Aniridia	Sporadic	PAX6	c.764A>G^a^ p.Gln255Arg	chr11-31815581-T-C
A115	Aniridia	Sporadic	PAX6	c.158T>C^a^ p.Val53Ala	chr11-31823308-A-G
A116	Aniridia	Sporadic	PAX6	c.551del p.Gly184Glufs*23	chr11-31816309-C-
A117 A241	Aniridia	Sporadic	PAX6	c.781C>T p.Arg261*	chr11-31815335-G-A
A118	Aniridia	Sporadic	PAX6	c.183C>A p.Tyr61*	chr11-31823283-G-T
A119	Aniridia	Sporadic	PAX6	c.949C>T p.Arg317*	chr11-31815069-G-A
A122	Aniridia	Familial	PAX6, ELP4	-^d^	arr[GRCh37]11p13(31642266_31825698)x1
A123	WAGR	Sporadic	EIF3M, ELP4, PAX6, RCN1, WT1	-	arr[GRCh37]11p13(31808455_32617592)x1
A125	Aniridia	Sporadic	PAX6	c.607C>T p.Arg203*	chr11-31816253-G-A
A130	Aniridia	Sporadic	PAX6	c.114_117del^a^ p.Pro39Alafs*14	chr11-31824276-CGGC-
A131	Aniridia	Sporadic	ELP4	c.1143+14176C>A^b^	chr11-31685945-C-A
A136 A146	Aniridia	Familial	PAX6	c.357+1G>A p.?	chr11-31823108-C-T
A137	WAGR	Sporadic	ABTB2, ANO3, APIP, ARL14EP, BBOX1, BDNF, CAPRIN1, CAT, CCDC34, CCDC73, CD44, CD59, CSTF3, DCDC1, DEPDC7, DNAJC24, EHF, EIF3M, ELF5, ELP4, FBXO3, FIBIN, FJX1, FSHB,HIPK3, IMMP1L, KCNA4, KIAA1549L, KIF18A, LGR4, LIN7C, LMO2, METTL15, MPPED2, MUC15, NAT10, PAMR1, PAX6, PDHX, PRRG4, QSER1, RCN1, SLC1A2, SLC5A12, TCP11L1, TRIM44, WT1	-^d^	arr[GRCh37]11p14.2p13(26583085_35710586)x1
A142	Aniridia	Sporadic	PAX6	c.765+1G>T p.?	chr11-31815579-C-A
A143	WAGR	Sporadic	ABTB2, ANO3, APIP, ARL14EP, BBOX1, BDNF, CAPRIN1, CAT, CCDC34, CCDC73, CD44, CD59, COMMD9, CSTF3, DCDC1, DEPDC7, DNAJC24, EHF, EIF3M, ELF5, ELP4, FBXO3, FIBIN, FJX1, FSHB, HIPK3, IFTAP, IMMP1L, KCNA4, KIAA1549L, KIF18A, LDLRAD3, LGR4, LIN7C, LMO2, METTL15, MPPED2, MUC15, NAT10, PAMR1, PAX6, PDHX, PRR5L, PRRG4, QSER1, RAG1, RAG2, RCN1, SLC1A2, SLC5A12, TCP11L1, TRAF6, TRIM44, WT1	-^d^	arr[GRCh37]11p14.3p12(25615512_39730342)x1
A151	Aniridia	Sporadic	PAX6	c.765+1G>C^a^ p.?	chr11-31815579-C-G
A152 A156B A156C	Aniridia	Familial	PAX6	c.358-2A>G p.?	chr11-31822406-T-C
A154 A257	Aniridia	Sporadic	PAX6	c.25_37del^a^ p.Asn9Valfs*18	chr11-31824356-CACCGAGCTGATT-
A165	Aniridia	Sporadic	PAX6	c.-128-2del p.?	chr11-31828475-T-
A168	Aniridia	Sporadic	PAX6	c.550G>T^a^ p.Gly184*	chr11-31816310-C-A
A172	Aniridia	Familial	DCDC1, DNAJC24, ELP4, IMMP1L	-^d^	arr[GRCh37]11p13(31176602_31714243)x1
A178 A179	Aniridia	Familial	PAX6	c.1068C>A^a^ p.Cys356*	chr11-31812373-G-T
A191	Aniridia	Familial	PAX6	c.1043_1056del^a^ p.Pro348Leufs*18	chr11-31812387-AGGTCTGGCTGGGG-
A192	Aniridia	Familial	PAX6	c.916+1G>A p.?	chr11-31815199-C-T
A204 A225	Aniridia	Familial	PAX6, ELP4	-^d^	arr[GRCh37]11p13(31540972_31813728)x1
A214	Aniridia	Sporadic	PAX6	c.1184-2A>G p.?	chr11-31811569-T-C
A215 A216	Rieger	Familial	PAX6	c.682+2T>C p.?	chr11-31816176-A-G
A223	Aniridia	Sporadic	PAX6	c.357+5G>A p.?	chr11-31823104-C-T
A226	Aniridia	Sporadic	PAX6	c.829C>T p.Gln277*	chr11-31815287-G-A
A232	Aniridia	Sporadic	PAX6	c.1268A>T p.*423Leuext*14	chr11-31811483-T-A
A240	Aniridia	Sporadic	PAX6	c.766-2A>T^a^ p.?	chr11-31815352-T-A
A242	Aniridia	Sporadic	PAX6	c.1A>G p.Met1Val	chr11-31827959-T-C
A245	Aniridia	Sporadic	PAX6	c.120C>A p.Cys40*	chr11-31824273-G-T
A247	Aniridia	Familial	PAX6	c.183delC p.Tyr61*	chr11-31823283-G-
A259 A270 A271	Aniridia	Familial	PAX6	c.859_862dup^a^ p.Ser288Asnfs*4	chr11-31815254--TGAT
A260 A262 A269	Aniridia	Familial	PAX6	c.1265del p.Gln422Argfs*103	chr11-31811486-T-
A263	Aniridia	Sporadic	PAX6	c.111_121delinsT^a^ p.Arg38Thrfs*13	chr11-31824272-CGCACGGCCGG-A
A264	Aniridia	Sporadic	PAX6	c.109del p.Ala37Profs*17	chr11-31824284-C-
A265	Aniridia	Sporadic	PAX6	c.433_443del^a^ p.Lys145Valfs*51	chr11-31822319-ATCCTTAGTTT-
A266	Aniridia	Sporadic	PAX6	c.1183G>A p.Gly395Arg	chr11-31812258-C-T
A274	Aniridia	Sporadic	PAX6	c.763C>T p.Gln255*	chr11-31815582-G-A
A276	Aniridia	Sporadic	PAX6	c.749_763del^a^ p.Pro250_Ile254del	chr11-31815582-GTATTCTTGCTTCAG-

^a^Variants not included in either ClinVar or HGMD databases. All variants refer to the RefSeq transcript ID NM_000280.5

^b^Previously reported in Bhatia et al. [19]

^c^Previously reported in Graziano et al. [12]

^d^Previously reported in Franzoni et al. [13]

### Variants of unknown significance in the 11p13 locus

Two variants of unknown significance (VUS) were found. A deletion involving most of the coding region of the *ELP4* gene, from intron 2–3 to intron 9–10 (arr[GRCh37]11p13(31541660_31802443)x1), was assessed in a patient with bilateral aniridia, nystagmus, posterior polar cataract, and bilateral corneal dystrophy (A114). Genetic counseling highlighted that her father shared the same phenotype but was unavailable for testing. The deletion removes diverse *PAX6* CREs including the ultra-conserved SIMO but does not entirely erase the critical region required for *PAX6* transcriptional activation described in Ansari et al. [20]. Recently, a smaller minimal critical region has been proposed, which includes four enhancers but spare SIMO [21]. For all these reasons, it cannot be considered definitely deleterious.

A heterozygous stop-loss variant in *PAX6* (NM_000280;c. 1267T > A;p.*423Lysext*14) was assessed in two unrelated patients both displaying aniridia (A205 and A273). The mutation abolished the canonical stop codon, generating a late stop codon after 42 base pairs, resulting in a 14 amino acid additional tail to the C-terminus of the PAX6 protein. This variant is located in a low-quality site within the gnomAD v2.1.1 database, therefore population frequency could not be considered. Indeed, both ClinVar and LOVD databases reported this variant as likely benign, contrary the current knowledge that PAX6 C-terminal extension variants recapitulate nonsense variants-related haploinsufficiency [18], but without experimental evidences. Given these assumptions, it was considered a VUS.

### Distribution of alterations in genes other than *PAX6*

Subsequently, WES was performed in *PAX6*-negative samples to identify rarer phenotypes or those phenotypes overlapping with aniridia, especially in the neonatal period (*n* = 64). After filtering benign and likely benign variants, 30.8% patients bore a pathogenic or likely pathogenic aberration in genes known to cause ocular dysgenesis. Both dominant and recessive patterns were assessed. Alteration in genes already related to ASD were found, such as a SNV in *PITX2*, four different *PXDN* missense variants in two unrelated patients, a nonsense *FZD5* variant in two related patients (A167, mother–A170, son), two variants in the recently discovered *MAB21L1* [22], and three *ITPR1* alterations, one in homozygous and two in heterozygous state.

Besides, aberrations in genes associated with complex syndromic phenotypes have been highlighted.

A heterozygous 5.2 Mb deletion involving chromosome X was found in a proband evaluated for suspected unilateral aniridia in the context of congenital corneal opacity (A84). Xp22.2 deletions are associated with the MIDAS syndrome, a rare syndromic eye disorder characterized by ocular defects and linear skin dysplasia [23]. Indeed, the patient’s phenotype evolved with unilateral sclerocornea and ptosis.

Two missense variants in the *CAPN15* gene were assessed in a patient displaying irido-choroidal coloboma associated with a mild developmental delay (A106). Biallelic *CAPN15* variants are associated to the oculogastrointestinal-neurodevelopmental syndrome (OMIM #619318), firstly reported in 2020 by Zha et al. [24]. Missense variants are known to be exclusively related to microphthalmia and/or coloboma [25].

The p.Arg179His hotspot variant in ACTA2 was identified in a patient with bilateral aniridia (A141). This variant is associated to the so-called multisystemic smooth muscle dysfunction syndrome (OMIM #613834), a rare vascular disease characterized by congenital mydriasis, patent ductus arteriosus, pulmonary artery hypertension, cerebrovascular disease, aortic anomalies, intestinal hypoperistalsis, and hypotonic bladder [26].

A *TFAP2A* missense variant was identified in a patient affected by chorioretinal and optic nerve coloboma (A153). *TFAP2A* monoallelic variants are associated to the branchio-oculo-facial syndrome (BOFS; OMIM #113620), characterized by branchial cleft sinus defects, ocular anomalies, and cleft or pseudocleft lip/palate [27]. BOFS is a distinctive and rare condition; the presence of phenotypic heterogeneity associated with specific genetic variants is still to be investigated.

Autosomal dominant tubulinopathy-associated dysgyria was diagnosed in a patient displaying a complex and severe phenotype which includes symptomatic focal epilepsy, tetraparesis, and cognitive impairment with absence of speech, micro-brachycephaly, dysmorphic features, convergent strabismus, eyelid ptosis, morning glory anomaly, in complex brain malformation (partial agenesis of the corpus callosum, hypoplasia of the cerebellar vermis, hypoplasia of the pons and midbrain) (A183). *TUBA1A* mutations are associated with severe congenital lissencephaly, due to abnormal neuronal migration involving neocortical and hippocampal lamination, corpus callosum, cerebellum, and brainstem [28]. The p.Arg390His variant identified in our patients has been associated to a milder TUBA1A phenotype, with dysplasia of the superior cerebellum, brainstem asymmetry, dysplasia of the basal ganglia, and cortical irregularities, but without pachygyria or polymicrogyria [29].

Lastly, a double molecular diagnosis was made in a patient affected by iris coloboma, psychomotor delay, and short stature (<3°p) (A237). A pathogenic heterozygous *PTPN11* variant and a 1.3 Mb deletion in 11q25 were assessed. *PTPN11* variants are associated to the Noonan syndrome, a well-known RASopathy characterized by short stature, facial dysmorphism, and a wide spectrum of congenital heart defects. Terminal 11q deletions are, instead, associated to the Jacobsen syndrome (JBS, OMIM #47791), whose phenotype may vary depending on the size of the deletion. Indeed, both conditions may be responsible for the occurrence of coloboma.

These findings are summarized in Table 2.

**Table 2:** Details of pathogenic or likely pathogenic alterations found by WES in our cohort

ID	Phenotype	Inheritance	Gene (transcript)	Variant	Genomic position (hg19)
A25	Peters anomaly	Sporadic	PXDN (NM_012293.3)	c.970G>T^a^ p.Gly324* c.1357C>T^a^ p.Gln453*	chr2-1677463-C-A chr2-1668781-G-A
A68	Aniridia, cataract, glaucoma and bufthalmos	Sporadic	PITX2 (NM_000325.6)	c.416G>C^a^ p.Trp139Ser	chr4-111539840-C-G
A84	Suspected aniridia, corneal opacity	Sporadic	AMELX, ARHGAP6, ATXN3L, CLCN4, CLDN34, EGFL6, FAM9C, FRMPD4, GEMIN8, GLRA2, GPM6B, GPR143, HCCS, MID1, MSL3, OFD1, PRPS2, RAB9A, SHROOM2, TBL1X, TCEANC, TLR7, TLR8, TMSB4X, TRAPPC2, WWC3	-	arr[GRCh37]Xp22.2(9651809_14770691)x1
A97	Bilateral partial aniridia, microphthalmia	Sporadic	MAB21L1 (NM_005584.5)	c.155T>G^b^ p.Phe52Cys	chr13-36050121-A-C
A106	Irido-choroidal coloboma, mild developmental delay	Sporadic	CAPN15 (NM_005632.3)	c.2207G>A^a^ p.Arg736Gln c.2352C>A^a^ p.Phe784Leu	chr16-601526-G-A + chr16-602057-C-A
A133	Bilateral microphthalmia, bilateral coloboma and unilateral optic nerve hypoplasia	Sporadic	PXDN (NM_012293.3)	c.562C>T^a^ p.Arg188* c.3614A>G^a^ p.Tyr1205Cys	chr2-1684133-G-A + chr2-1648519-T-C
A141	Bilateral aniridia	Sporadic	ACTA2 (NM_001613.4)	c.536G>A p.Arg179His	chr10-90701066-C-T
A153	Chorioretinal and optic nerve coloboma, microcornea	Sporadic	TFAP2A (NM_001372066.1)	c.1039T>C p.Cys347Arg	chr6-10398698-A-G
A167 A170	Coloboma	Familial	FZD5 (NM_003468.4)	c.236C>A^a^ p.Ser79*	chr2-207768504-G-T
A180	Bilateral aniridia, microphthalmia, microcornea, nystagmus, mild bilateral optic nerve hypoplasia	Sporadic	MAB21L1 (NM_005584.5)	c.152G>A p.Arg51Gln	chr13-36050124-C-T
A183	Morning glory anomaly, strabismus epilepsy, dysmorphic features	Sporadic	TUBA1A (NM_006009.4)	c.1169G>A p.Arg390His	chr12-49578980-C-T
A210	Aniridia, intellectual disability	Familial	ITPR1 (NM_001378452.1)	c.279+4_279+7delCGTA (homozygous) p.?	chr3-4669561-ACGT-
A237	Iris coloboma, psychomotor delay, short stature	Sporadic	PTPN11 (NM_002834.5) ACAD8, B3GAT1, GLB1L2, GLB1L3, IGSF9B, JAM3, NCAPD3, SPATA19, THYN1, VPS26B	c.1508G>A p.Gly503Glu -	chr12-112926888-G-A arr[GRCh37]11q25(133711934_135006515)x1
A244	Aniridia, glaucoma, nystagmus, corneal opacity	Sporadic	CYP1B1 (NM_000104.4)	c.352C>T p.Pro118Ser c.1064_1076delGAGTGCAGGCAGA p.Arg355Hisfs*69	chr2-38302180-G-A chr2-38298421-TCTGCCTGCACTC--
A253	Bilateral aniridia, nystagmus, scotopia psychomotor delay, hearing loss	Sporadic	ITPR1 (NM_001378452.1)	c.7660G>A p.Gly2554Arg	chr3-4856205-G-A
A258	Aniridia	Sporadic	ITPR1 (NM_001378452.1)	c.7666G>A^a^ p.Gly2556Arg	chr3-4856211-G-A

^a^Variants not included in either ClinVar or HGMD databases

^b^Published in Hall et al. [22]

### Variants of unknown significance identified by WES

Three VUSs were identified. A SNV in exon 1 of the *FOXC1* gene in two related patients (A44, mother–A45, daughter) displaying aniridia. This variant (NM_001453.3: c.1159G > C; p.Ala387Pro) was already reported in ClinVar associated with Axenfeld–Rieger syndrome. A SNV in *SOX2* (NM_003106.4:c.611C > T; p.Ala204Val) in a patient with Peters anomaly (A200). Recently, *SOX2* alterations have been associated with this condition [30]. Notwithstanding, this is the first time that this variant has been reported. Lastly, a 1.54 Mb deletion involving the *MAF* gene (arr[GRCh37]16q23.1q23.2(79030057_80574915)x1) was highlighted in a patient with bilateral aniridia (A256). Indeed, missense mutations in *MAF* are associated to both autosomal dominant cataract and the Ayme–Gripp syndrome. Our patients had no phenotypic overlap with both these conditions.

### Molecular diagnosis rates

Taken together, 77.2% of samples (*N* = 125) bore a positive genetic test, with the highest percentage considering aniridia patients only, both isolated and syndromic (89.9%). A VUS was found in 4.3% samples (*N* = 7) while a negative genetic test was assessed in 18.5% patients (*N* = 30). All these data are summarized in Supplementary Tables 2 and 3.

## Discussion

Phenotypic and genotypic heterogeneity, especially in ASD, have created challenges for proper diagnosis and classification of disease [3]. Genes involving both autosomal recessive and autosomal dominant patterns of inheritance have been described with a wide phenotypic variability and expressivity.

Indeed, the introduction of NGS has revolutionized the field of human genetics, increasing the opportunity to establish molecular diagnoses and identify new associated genes. The usefulness of genetic testing by NGS is manifold: (i) it provides a more precise diagnosis, especially in the neonatal period when the phenotype may not yet have fully manifested; (ii) it can be used for carrier screening, which widens the choice of reproductive options for those who are diagnosed as carriers, (iii) it could foster the development of novel treatments that are genotype specific [1]. Notwithstanding, genetic testing comes with great challenges mostly related to variant interpretation.

In the last 20 years, we collected a cohort of 162 patients affected by ocular dysgenesis, mostly aniridia.

**Fig. 4:**
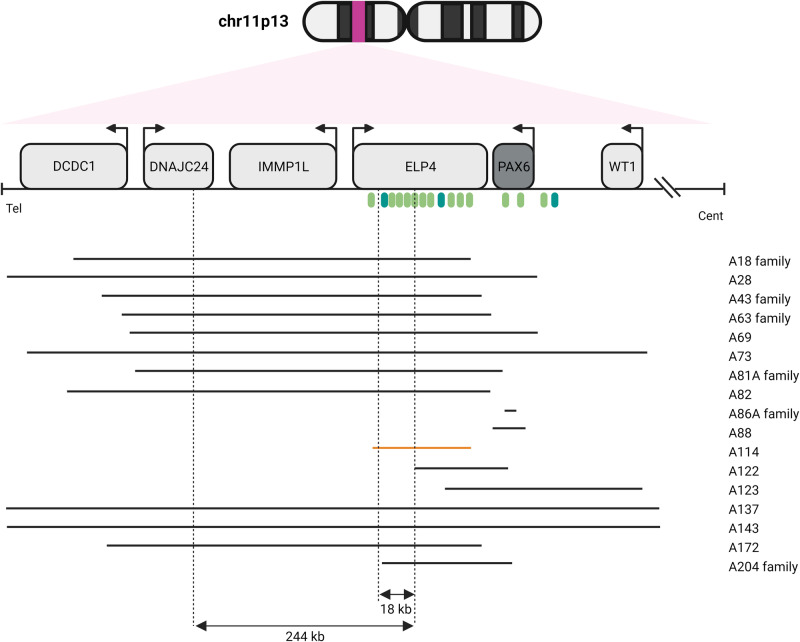
Schematic representation of 11p13 deletions assessed in our cohort. 3′ of PAX6. Genes are represented by light gray and gray boxes. Arrows indicate the direction of transcription. Light green eclipses represent enhancers. Dark green eclipses represent SIMO and E180B. Black lines represent pathogenic and likely pathogenic deletions found in our cohort, while the orange line represents the VUS. Vertical dashed lines represent the 244 kb and the 18 kb *PAX6* critical regulatory regions identified by [20] and [21], respectively. Some of these deletions are also described in [13]. Genomic coordinates are based on human genome assembly hg19

Our data certainly reiterate the notion that PAX6 alterations are primarily associated with ASD, since the majority of the cohort (66.7%) has a pathogenic or likely pathogenic variant in the *PAX6* locus. This percentage gets an obvious increase by analyzing only isolated or syndromic aniridia cases (82.2%). Indeed, previous literature clearly demonstrated that *PAX6* alterations explain about 80% of aniridia patients, both sporadic and familial [31]. The landscape of *PAX6* coding variants is similar to that reported in literature [18, 32] even though we assessed a small augmentation in splice site variants (25.4% vs 15%). Exon 5 is proven to include the largest number of variants, a foreseeable event given that exons 5 and 6 encode for the paired domain, one of the PAX6 DNA-binding domains that has critical roles in development of the eye, the pancreas, and the central nervous system [33]. What proves to be worthy of attention is that our cohort included a striking fraction of deletions involving the 11p13 locus (20.8% of all *PAX6*-positive samples), either partially/totally involving *PAX6* coding region or abolishing its critical regulatory region (Fig. 4, Table 1). *PAX6* is surrounded by CREs that spatially and temporally direct its expression at different developmental stages. The large *PAX6* regulatory landscape contains several enhancers that act in a finely controlled manner to direct *PAX6* expression in the developing central nervous system, retina, lens, olfactory bulb, and pancreas [16, 17, 34].

The regulatory role of these CREs was suggested by the existence of aniridia in patients with different chromosome 11p13 rearrangements affecting the downstream elements while preserving the *PAX6* coding sequence [21, 35]. Therefore, over the past 15 years a significant part of the ophthalmologic research has focused on characterizing these CREs. Given the abundance of regulatory sequences surrounding PAX6, some amount of overlap among the regulatory sequences in directing PAX6 in specific tissues has always been assumed [16]. Indeed whether these enhancers perform additive, redundant, or distinct functions is largely unknown. Studies in zebrafish have allowed to recognize specific spatio-temporal activity patterns for some regulatory sequences [36, 37]. Uttley et al. demonstrated using dual enhancer–reporter zebrafish embryos that the two PAX6 retinal enhancers HS5 and NRE have both overlapping and spatio-temporal specific activities [37]. Besides, with an exception in the SIMO lens enhancer, deleterious point mutation affecting these CREs appears to be an ultra-rare event. All these assumptions emphasize how little we actually know about how these enhancers work in regulating *PAX6* expression. Furthermore, which is the minimal regulatory region that if deleted is able to elicit aniridia is still debated. To date, a large number of 11p13 deletions have been already identified [21, 38, 39], but correlation with phenotypes is still scanty. In 2016, Ansari et al. postulated a 244 kb critical region required for *PAX6* transcriptional activation [20]. From this time forward, several papers assessed smaller deletions, partially involving *ELP4*, in patients affected by aniridia [21, 40].

This turns out to be of particular interest by analyzing one of the VUS identified in this cohort, i.e., a 260.8 kb deletion assessed in a patient with familial aniridia (A114) involving part of the *ELP4* gene (int2-3 to int9-10). The deletion removes at least 10 *PAX6* CREs including RB, E180B, HS2-8, E100, E120, SIMO, and E60 [19, 21], but does not entirely erase the 244 kb critical region proposed by Ansari et al. [20]. Plaisanciè et al. proposed an 18 kb minimal region including the E180B enhancer that, if deleted, correlates with the aniridia phenotype without extraocular manifestations. Our deletion removes an interaction-rich region toward both 3′ and 5′, which includes both SIMO and E180B, so its involvement in the patient’s phenotype would not be surprising. However, these are assumptions that will have to be validated by future studies, perhaps in vivo.

The use of WES has allowed us to expand the analytical portfolio and identify rarer phenotypes. Indeed, multiple conditions and syndromes are categorized under the umbrella of ASD [41], and nowadays at least 60 genes have been associated with this condition [5]. SNVs in genes strongly associated with ocular dysgenesis have been found in our cohort, including *ACTA2*, *CYP1B1*, *FZD5*, *PXDN*, *PITX2*, and *TFAP2A*. Besides *PAX6*, the second most altered gene in our cohort turned out to be *ITPR1*, whose either homozygous or heterozygous, dominant-negative, pathogenic variants are associated to the Gillespie syndrome—characterized by a triad of partial aniridia, non-progressive cerebellar ataxia, and intellectual disability [42]. Gillespie syndrome is an exceptionally uncommon diagnosis with <50 patients ever being diagnosed [43]. None of our patients (A210, A253, and A258) presented the classical triad of symptoms, with two out of three patients affected by intellectual disability. No signs of ataxia were detected. This is not surprising given that papers describing patients with atypical presentations have been only recently published [43]. Furthermore, two *MAB21L1* variants were found in our cohort, the hotspot mutation p.Arg51Leu and the previously published p.Phe52Cys (A180 and A97, respectively) [22]. Indeed, *MAB21L1* is gaining momentum as a novel gene associated with severe aniridia and/or microphthalmia [22, 44].

Furthermore, WES allowed us to diagnose severe syndromic conditions such as the MIDAS syndrome, the oculogastrointestinal-neurodevelopmental syndrome and the JBS. MIDAS (microphthalmia, dermal aplasia, and sclerocornea) syndrome has been described 30 years ago. Ocular findings commonly include microphthalmia and sclerocornea even though corneal opacities without sclerocornea, microcornea, corneal leukoma, congenital glaucoma, aniridia, cataract, and Peters’ anomaly have been described. It is caused by Xp22.2 deletions and it is characterized by wide inter- and intra-familial phenotypic variability, which has been associated with skewed X inactivation of the genes involved [45]. Concerning the proband (A84), skin defects were not reported at birth. Some unusual MIDAS presentations could be assessed in literature, with eye abnormalities in the absence of skin defects and vice versa [23].

To date, oculogastrointestinal-neurodevelopmental syndrome has been described in less than ten published individuals, and it is associated with biallelic pathogenic variants in the *CAPN15* gene [25]. While loss of function variants are associated to a more severe phenotype including ocular defects, microcephaly, craniofacial abnormalities, cardiac and genitourinary malformations, abnormal neurologic activity, and developmental delay, missense variants rise a milder phenotype mostly characterized by microphthalmia and/or coloboma in association with mild developmental delay [46]. All this is in accordance with our patient’s phenotype (A106).

Lastly, the JBS is a rare and poorly understood multisystem genomic disorder where the distal region of chromosome 11q is deleted. It is a quite rare condition characterized by multiple anomalies including developmental delay, craniofacial dysmorphisms, craniosynostosis, ocular abnormalities, congenital heart disease, intellectual disability, Paris Trousseau hemorrhagic disease, and immunodeficiency [47]. Notwithstanding, clinical manifestations depend on the size of deletion, which usually varies between 7 and 20 Mb [48]. Our patient (A237) bore a 1.2 Mb deletion involving only the 11q25 cytoband, which probably does not result in a full-blown JBS phenotype. Indeed, the 11q24 locus has been associated with the thrombocytopenia and Paris Trousseau hemorrhagic disease, typical features of this disorder [49]. Moreover, the clinical picture of our patient is complicated by the association of the partial JBS phenotype and an already known pathogenic variant in the *PTPN11* gene. RASopathies are a group of autosomal dominant disorders caused by pathogenic variants in genes encoding proteins of the RAS/mitogen-activated protein kinase pathway, such as *PTPN11*. The clinical spectrum is characterized by specific facial features, congenital heart disease (CHD), and hypertrophic cardiomyopathy (HCM), and variable postnatal growth retardation, neurological involvement, and cancer predisposition [50]. Recently ocular coloboma was assessed in a 7 y.o. patient with a genetically proven Noonan syndrome due to a *PTPN11* mutation [51].

A small number of VUS have been also identified, such as a 1.54 Mb deletion in the 16q23.1q23.2 locus. The deletion partially removes *WWOX* coding region (intron 8–9 to 5′UTR), whose biallelic variants are known to cause a severe early-onset epileptic encephalopathy. No other variants affecting this gene were assessed in our patient (A256). More interesting turns out to be the involvement of the *MAF* gene, which is found to be completely included in the deletion. Missense mutation in *MAF* are associated to both autosomal dominant cataract and the Ayme–Gripp syndrome, a clinically homogeneous disorder characterized by congenital cataracts, sensorineural hearing loss, intellectual disability, seizures, brachycephaly, a distinctive flat facial appearance, and reduced growth [52]. Indeed, our patient had no phenotypic overlap with both these conditions and was diagnosed with bilateral aniridia. The *MAF bZIP transcription factor* (*MAF*) is an important regulator of eye development, specifically lens development [53]. *MAF* point mutations have been associated with ocular malformations, such as iris coloboma, congenital cataract, glaucoma, and microphthalmia [54]. To date, *MAF* whole gene deletions have never been correlated to aniridia. Further experiments needed to be performed to clearly validate this novel association.

Comparing our data with other studies in this field published in the past 5 years, it is evident that the composition of the cohorts analyzed is quite variable, as are the molecular technologies applied. Cross et al. applied both direct sequencing and MLPA in a cohort of 434 subjects undergoing diagnostic testing for PAX6 [55]. Vasilyeva et al., who screened 199 patients for variants at the 11p13 locus, adopted the same analytical approach [56]. In these works, the diagnostic rate is very different, 58.5% and 69%, respectively, and this is due to the different composition of the cohort (59% vs 92% of patients with classical aniridia). When less “narrow” technological approaches were employed, the number of patients analyzed was reduced with a significant decrease in diagnostic yield. Targeted sequencing, WES and WGS were performed with an average of 47% positive molecular diagnoses [11, 30, 57, 58].

In conclusion, our data certainly reiterate the notion that *PAX6* alterations are primarily associated with the development of isolated aniridia. It is worth of attention that 20.3% of unique causative variants consist in deletions involving chromosome 11p13, one-third of which exclusively involve *PAX6* regulatory regions. The spectrum of deletions identified in this study also enables us to expand the knowledge of how little we still know about the minimal critical region capable of altering eye development. Furthermore, the distinctive enrichment of deletions, most of which involve familial cases, is not due to a marked representation of WAGR cases (only four in the entire cohort) but probably to the size of the cohort itself. Notwithstanding, given the increasing implication of alterations in the regulatory regions of PAX6 eliciting aniridia, a better understanding of PAX6 CREs and of the regulatory regions of other ASD-associated genes would ensure their analysis for diagnostic purposes.

To the best of our knowledge, our cohort is the only Italian cohort published so far. We suggest that the use of WES is critical for the differential diagnosis of those conditions in which there may be phenotypic overlap and in general in ASD, both isolated and syndromic. Indeed, our study has allowed us to identify rare conditions with ocular involvement that contribute to a better understanding of the molecular pathways underlying ocular development.

Lastly, a small percentage of patients still test negative suggesting that: (i) complex rearrangements (i.e., inversions) or deep intronic variants are putatively involved in ocular dysgenesis; (ii) there is room for research into new ultra-rare disease genes.

### Supplementary information

Supplementary Table 1

Supplementary Table 2

Supplementary Table 3

## Data Availability

The ethics committee approval does not allow sharing of complete, raw data from NGS or the full list of variants. Notwithstanding, data are available from the corresponding author on reasonable request.

## References

[1] Verma IC, Paliwal P, Singh K (2018). Genetic testing in pediatric ophthalmology. Indian J Pediatr.

[2] Guercio JR, Martyn LJ (2007). Congenital malformations of the eye and orbit. Otolaryngol Clin N Am.

[3] Ito YA, Walter MA (2014). Genomics and anterior segment dysgenesis: a review. Clin Exp Ophthalmol.

[4] Villalba MF, Li CM, Pakravan P, Bademci G, Chang TCP (2023). Commercial gene panels for congenital anterior segment anomalies: are they all the same?. Am J Ophthalmol.

[5] Ma A, Yousoof S, Grigg JR, Flaherty M, Minoche AE, Cowley MJ (2020). Revealing hidden genetic diagnoses in the ocular anterior segment disorders. Genet Med.

[6] Kaushik S, Dubey S, Choudhary S, Ratna R, Pandav SS, Khan AO (2022). Anterior segment dysgenesis: insights into the genetics and pathogenesis. Indian J Ophthalmol.

[7] Arte S, Pöyhönen M, Myllymäki E, Ronkainen E, Rice DP, Nieminen P (2023). Craniofacial and dental features of Axenfeld–Rieger syndrome patients with PITX2 mutations. Orthod Craniofac Res.

[8] Qiao Y, Chen Y, Tan C, Sun X, Chen X, Chen J (2021). Screening and functional analysis of TEK mutations in Chinese children with primary congenital glaucoma. Front Genet.

[9] Yu-Wai-Man C, Arno G, Brookes J, Garcia-Feijoo J, Khaw PT, Moosajee M (2018). Primary congenital glaucoma including next-generation sequencing-based approaches: clinical utility gene card. Eur J Hum Genet.

[10] Stingl JV, Diederich S, Diel H, Schuster AK, Wagner FM, Chronopoulos P (2021). First results from the prospective German registry for childhood glaucoma: phenotype–genotype association. J Clin Med.

[11] Haug P, Koller S, Maggi J, Lang E, Feil S, Wlodarczyk A (2021). Whole exome sequencing in coloboma/microphthalmia: identification of novel and recurrent variants in seven genes. Genes.

[12] Graziano C, D’Elia AV, Mazzanti L, Moscano F, Guidelli Guidi S, Scarano E (2007). A de novo nonsense mutation of PAX6 gene in a patient with aniridia, ataxia, and mental retardation. Am J Med Genet A.

[13] Franzoni A, Russo PD, Baldan F, D’Elia AV, Puppin C, Penco S (2017). A CGH array procedure to detect PAX6 gene structural defects. Mol Cell Probes.

[14] Codrich M, Dalla E, Mio C, Antoniali G, Malfatti MC, Marzinotto S (2021). Integrated multi-omics analyses on patient-derived CRC organoids highlight altered molecular pathways in colorectal cancer progression involving PTEN. J Exp Clin Cancer Res.

[15] Richards S, Aziz N, Bale S, Bick D, Das S, Gastier-Foster J (2015). Standards and guidelines for the interpretation of sequence variants: a joint consensus recommendation of the American College of Medical Genetics and Genomics and the Association for Molecular Pathology. Genet Med.

[16] McBride DJ, Buckle A, van Heyningen V, Kleinjan DA. DNaseI hypersensitivity and ultraconservation reveal novel, interdependent long-range enhancers at the complex Pax6 cis-regulatory region. PLoS ONE. 2011;6. http://www.ncbi.nlm.nih.gov/pmc/articles/PMC3248410/. 10.1371/journal.pone.0028616 PMC3248410 22220192

[17] Bhatia S, Monahan J, Ravi V, Gautier P, Murdoch E, Brenner S (2014). A survey of ancient conserved non-coding elements in the PAX6 locus reveals a landscape of interdigitated cis-regulatory archipelagos. Dev Biol.

[18] Lima Cunha D, Arno G, Corton M, Moosajee M (2019). The spectrum of PAX6 mutations and genotype–phenotype correlations in the eye. Genes.

[19] Bhatia S, Bengani H, Fish M, Brown A, Divizia MT, de Marco R (2013). Disruption of autoregulatory feedback by a mutation in a remote, ultraconserved PAX6 enhancer causes aniridia. Am J Hum Genet.

[20] Ansari M, Rainger J, Hanson IM, Williamson KA, Sharkey F, Harewood L, et al. Genetic analysis of ‘PAX6-negative’ individuals with aniridia or Gillespie syndrome. PLoS ONE. 2016;11. http://www.ncbi.nlm.nih.gov/pmc/articles/PMC4849793/. 10.1371/journal.pone.0153757 PMC4849793 27124303

[21] Plaisancié J, Tarilonte M, Ramos P, Jeanton-Scaramouche C, Gaston V, Dollfus H (2018). Implication of non-coding PAX6 mutations in aniridia. Hum Genet.

[22] Hall HN, Bengani H, Hufnagel RB, Damante G, Ansari M, Marsh JA (2022). Monoallelic variants resulting in substitutions of MAB21L1 Arg51 cause aniridia and microphthalmia. PLoS ONE.

[23] Franco E, Scanga HL, Nischal KK (2023). Variable phenotype of secondary congenital corneal opacities associated with microphthalmia with linear skin defects syndrome. Am J Med Genet A.

[24] Zha C, Farah CA, Holt RJ, Ceroni F, Al-Abdi L, Thuriot F (2020). Biallelic variants in the small optic lobe calpain CAPN15 are associated with congenital eye anomalies, deafness and other neurodevelopmental deficits. Hum Mol Genet.

[25] Beaman MM, Guidugli L, Hammer M, Barrows C, Gregor A, Lee S (2023). Novel association of Dandy–Walker malformation with CAPN15 variants expands the phenotype of oculogastrointestinal neurodevelopmental syndrome. Am J Med Genet A.

[26] Yang WX, Zhang HH, Hu JN, Zhao L, Li YY, Shao XL (2021). ACTA2 mutation is responsible for multisystemic smooth muscle dysfunction syndrome with seizures: a case report and review of literature. World J Clin Cases.

[27] Min J, Mao B, Wang Y, He X, Gao S, Wang H (2020). A heterozygous novel mutation in TFAP2A gene causes atypical branchio-oculo-facial syndrome with isolated coloboma of choroid: a case report. Front Pediatr.

[28] Tischfield MA, Cederquist GY, Gupta ML, Engle EC (2011). Phenotypic spectrum of the tubulin-related disorders and functional implications of disease-causing mutations. Curr Opin Genet Dev..

[29] Zanni G, Colafati GS, Barresi S, Randisi F, Talamanca LF, Genovese E (2013). Description of a novel TUBA1A mutation in Arg-390 associated with asymmetrical polymicrogyria and mid-hindbrain dysgenesis. Eur J Paediatr Neurol.

[30] Chesneau B, Aubert-Mucca M, Fremont F, Pechmeja J, Soler V, Isidor B (2022). First evidence of SOX2 mutations in Peters’ anomaly: lessons from molecular screening of 95 patients. Clin Genet.

[31] Grønskov K, Olsen JH, Sand A, Pedersen W, Carlsen N, Jylling A (2001). Population-based risk estimates of Wilms tumor in sporadic aniridia. Hum Genet.

[32] Prosser J, van Heyningen V (1998). PAX6 mutations reviewed. Hum Mutat.

[33] Linkroum K, DelBono EA, Wiggs JL (2010). A novel Pax6 in-frame insertion in the paired domain is associated with a severe ocular phenotype. Investig Ophthalmol Vis Sci.

[34] Buckle A, Nozawa Rsuke, Kleinjan DA, Gilbert N (2018). Functional characteristics of novel pancreatic Pax6 regulatory elements. Hum Mol Genet.

[35] Fantes J, Redeker B, Breen M, Boyle S, Brown J, Fletcher J (1995). Aniridia-associated cytogenetic rearrangements suggest that a position effect may cause the mutant phenotype. Hum Mol Genet.

[36] Damián A, Núñez-Moreno G, Jubin C, Tamayo A, de Alba MR, Villaverde C (2023). Long-read genome sequencing identifies cryptic structural variants in congenital aniridia cases. Hum Genom.

[37] Uttley K, Papanastasiou AS, Lahne M, Brisbane JM, MacDonald RB, Bickmore WA (2023). Unique activities of two overlapping PAX6 retinal enhancers. Life Sci Alliance.

[38] Simioni M, Vieira TP, Sgardioli IC, Freitas ÉL, Rosenberg C, Maurer-Morelli CV (2012). Insertional translocation of 15q25–q26 into 11p13 and duplication at 8p23.1 characterized by high resolution arrays in a boy with congenital malformations and aniridia. Am J Med Genet A..

[39] Addis L, Ahn JW, Dobson R, Dixit A, Ogilvie CM, Pinto D (2015). Microdeletions of ELP4 are associated with language impairment, autism spectrum disorder, and mental retardation. Hum Mutat.

[40] Blanco-Kelly F, Palomares M, Vallespín E, Villaverde C, Martín-Arenas R, Vélez-Monsalve C (2017). Improving molecular diagnosis of aniridia and WAGR syndrome using customized targeted array-based CGH. PLoS ONE.

[41] Zhu AY, Costain G, Cytrynbaum C, Weksberg R, Cohn RD, Ali A (2021). Novel heterozygous variants in PXDN cause different anterior segment dysgenesis phenotypes in monozygotic twins. Ophthalmic Genet.

[42] Keehan L, Jiang MM, Li X, Marom R, Dai H, Murdock D (2021). A novel de novo intronic variant in ITPR1 causes Gillespie syndrome. Am J Med Genet A.

[43] Singh G, Narahari S (2022). A case of Gillespie syndrome with atypical presentation. Cureus.

[44] Wang P, Wu P, Wang J, Zeng Y, Jiang Y, Wang Y (2023). Missense mutations in MAB21L1: causation of novel autosomal dominant ocular BAMD syndrome. Investig Ophthalmol Vis Sci.

[45] Indrieri A, Franco B (2021). Linear skin defects with multiple congenital anomalies (LSDMCA): an unconventional mitochondrial disorder. Genes.

[46] Mor-Shaked H, Salah S, Yanovsky-Dagan S, Meiner V, Atawneh OM, Abu-Libdeh B (2021). Biallelic deletion in a minimal CAPN15 intron in siblings with a recognizable syndrome of congenital malformations and developmental delay. Clin Genet.

[47] Gunaseelan S, Wang Z, Tong VKJ, Ming SWS, Razar RBBA, Srimasorn S (2021). Loss of FEZ1, a gene deleted in Jacobsen syndrome, causes locomotion defects and early mortality by impairing motor neuron development. Hum Mol Genet.

[48] Bernaciak J, Szczałuba K, Derwińska K, Wiśniowiecka-Kowalnik B, Bocian E, Sąsiadek MM (2008). Clinical and molecular-cytogenetic evaluation of a family with partial Jacobsen syndrome without thrombocytopenia caused by an ∼5 Mb deletion del(11)(q24.3). Am J Med Genet A.

[49] Tyson C, Qiao Y, Harvard C, Liu X, Bernier FP, McGillivray B (2008). Submicroscopic deletions of 11q24-25 in individuals without Jacobsen syndrome: re-examination of the critical region by high-resolution array-CGH. Mol Cytogenet.

[50] Leoni C, Blandino R, Delogu AB, De Rosa G, Onesimo R, Verusio V (2022). Genotype-cardiac phenotype correlations in a large single-center cohort of patients affected by RASopathies: clinical implications and literature review. Am J Med Genet A.

[51] Van den Heurck JJ, Boven KB, Claes CC (2023). Optic disk coloboma and contralateral optic disk pit maculopathy treated by vitrectomy in a patient with Noonan syndrome with multiple lentigines. Retin Cases Brief Rep.

[52] Niceta M, Stellacci E, Gripp KW, Zampino G, Kousi M, Anselmi M (2015). Mutations impairing GSK3-mediated MAF phosphorylation cause cataract, deafness, intellectual disability, seizures, and a Down syndrome-like facies. Am J Hum Genet.

[53] Kinoshita A, Ohyama K, Tanimura S, Matsuda K, Kishino T, Negishi Y (2021). Itpr1 regulates the formation of anterior eye segment tissues derived from neural crest cells. Development.

[54] Anand D, Agrawal SA, Slavotinek A, Lachke SA (2018). Mutation update of transcription factor genes FOXE3, HSF4, MAF, and PITX3 causing cataracts and other developmental ocular defects. Hum Mutat.

[55] Cross E, Duncan-Flavell PJ, Howarth RJ, Crooks RO, Thomas NS, Bunyan DJ (2020). Screening of a large PAX6 cohort identified many novel variants and emphasises the importance of the paired and homeobox domains. Eur J Med Genet.

[56] Vasilyeva TA, Marakhonov AV, Kutsev SI, Zinchenko RA (2022). Relative frequencies of PAX6 mutational events in a Russian cohort of aniridia patients in comparison with the world’s population and the human genome. Int J Mol Sci.

[57] Jackson D, Malka S, Harding P, Palma J, Dunbar H, Moosajee M (2020). Molecular diagnostic challenges for non-retinal developmental eye disorders in the United Kingdom. Am J Med Genet C Semin Med Genet.

[58] Lenassi E, Clayton-Smith J, Douzgou S, Ramsden SC, Ingram S, Hall G (2020). Clinical utility of genetic testing in 201 preschool children with inherited eye disorders. Genet Med.

