# Supplementary material for: A bird’s eye view on the use of whole exome sequencing in rare congenital ophthalmic diseases
Source: J Hum Genet. 2024 Mar 8;69(6):271–82. doi: 10.1038/s10038-024-01237-6 (PMC11126393; doi:10.1038/s10038-024-01237-6)
Supplement: Supplementary file 1 — Supplementary Table 1 [file 10038_2024_1237_MOESM1_ESM.docx]

**Supplementary Table 1. Clinical features, genetic alterations and inheritance of ASD.**

| Disease | HPO term | Prevalence | Main clinical characteristics | Inheritance | Genes involved | References |
| --- | --- | --- | --- | --- | --- | --- |
| Aniridia | HP:0000526 | 1:50’000–100’000  live births | Various degrees of iris hypoplasia, variable foveal hypoplasia, ectopia lentis, keratopathy, glaucoma, cataract, dry eye disease. | Autosomal dominant | *FOXC1*, *PAX6*, *TRIM44* | (1–4) |
| Anophthalmia microphthalmia and coloboma (MAC) | HP:0000528  HP:0000568  HP:0000589 | 3-30:100’000  live births | Anophthalmia: complete absence of the eye.  Microphthalmia: small eye in terms of axial length and corneal diameter.  Coloboma: partial or complete failure of optic fissure closure. | Autosomal dominant  Autosomal recessive | >300 genes | (5–7) |
| Axenfeld-Rieger syndrome  (ARS) | HP:0000558 | 1:200’000  live births | Anterior segment dysgenesis; craniofacial, dental, umbilical, and cardiac anomalies | Autosomal dominant | *FOXC1*, *PITX2*, *RIEG2*^¤^ | (8–10) |
| Congenital cataracts (CC) | HP:0000519 | 1-3:10’000  live births | Partial or complete lens opacification | Autosomal dominant  Autosomal recessive  X-linked | >350 genes | (11–14) |
| Gillespie syndrome (GS) | n.a. | <1:1’000’000  live births | Symmetric partial aplasia of the iris occurring with a fixed mydriasis, congenital hypotonia, cerebellar ataxia and variable degrees of intellectual disability | Autosomal dominant  Autosomal recessive | *ITPR1* | (15–17) |
| Morning glory anomaly (MGSA) | HP:0025514 | 2.6:100’000  live births | Enlarged, dysplastic and funnel-shaped optic nerve | n.a. | *PAX6* | (18–20) |
| Peters anomaly (PA) | HP:0000659 | <1:1’000’000  live births | Central corneal opacity, iridocorneal or lenticulo-corneal adhesions, glaucoma, microcornea, microphthalmos, cornea plana, sclerocornea, coloboma, aniridia, dysgenesis of the angle and iris and persistent hyperplastic primary vitreous | Autosomal dominant  Autosomal recessive | *B3GLCT*, *PAX6*, *PITX2*, *PITX3*, *FOXE3*, *FOXC1*, *COL4A1*, *CYP1B1*, *MAF* | (21–24) |
| Primary congenital glaucoma (PCG) | HP:0008007 | 3.4:100’000  live births | Elevated intraocular pressure, edema and opacification of the cornea, rupture of Descemet's membrane, iris atrophy, thinning of the anterior sclera, buphthalmos, anomalously deep anterior chamber, progressive glaucomatous retinal nerve fiber loss | Autosomal dominant  Autosomal recessive | *CYP1B1*, *LTBP2*, *MYOC*, *FOXC1*, *TEK*, *ANGPT1* | (25–29) |
| Wilms tumor-aniridia-genital anomalies-retardation (WAGR) | n.a. | 1:500’000–1’000’000  live births | Congenital developmental abnormalities, intellectual disability, increased risk of developing Wilms tumor | Autosomal dominant | *PAX6*, *WT1* | (1,3) |

^¤^ RIEG2: 13q14 locus associated to ARS in which the altered gene has not been yet identified.

**References**

1. Grønskov K, Olsen JH, Sand A, Pedersen W, Carlsen N, Jylling A, et al. Population-based risk estimates of Wilms tumor in sporadic aniridia. Hum Genet. 2001 Jul 1;109(1):11–8.

2. Hingorani M, Hanson I, van Heyningen V. Aniridia. Eur J Hum Genet. 2012 Oct;20(10):1011–7.

3. Blanco-Kelly F, Tarilonte M, Villamar M, Damián A, Tamayo A, Moreno-Pelayo MA, et al. Genetics and epidemiology of aniridia: Updated guidelines for genetic study. Archivos de la Sociedad Española de Oftalmología (English Edition). 2021 Nov 1;96:4–14.

4. Landsend ECS, Lagali N, Utheim TP. Congenital aniridia – A comprehensive review of clinical features and therapeutic approaches. Survey of Ophthalmology. 2021 Nov 1;66(6):1031–50.

5. Skalicky SE, White AJR, Grigg JR, Martin F, Smith J, Jones M, et al. Microphthalmia, Anophthalmia, and Coloboma and Associated Ocular and Systemic Features: Understanding the Spectrum. JAMA Ophthalmology. 2013 Dec 1;131(12):1517–24.

6. Zha C, Farah CA, Holt RJ, Ceroni F, Al-Abdi L, Thuriot F, et al. Biallelic variants in the small optic lobe calpain CAPN15 are associated with congenital eye anomalies, deafness and other neurodevelopmental deficits. Hum Mol Genet. 2020 Sep 3;29(18):3054–63.

7. Holt R, Goudie D, Verde AD, Gardham A, Ramond F, Putoux A, et al. Individuals with heterozygous variants in the Wnt-signalling pathway gene FZD5 delineate a phenotype characterized by isolated coloboma and variable expressivity. Ophthalmic Genetics. 2022 Nov 2;43(6):809–16.

8. Song W, Hu X. The rare Axenfeld–Rieger syndrome with systemic anomalies: A case report and brief review of literature. Medicine. 2017 Aug;96(33):e7791.

9. Arte S, Pöyhönen M, Myllymäki E, Ronkainen E, Rice DP, Nieminen P. Craniofacial and dental features of Axenfeld-Rieger syndrome patients with PITX2 mutations. Orthodontics & Craniofacial Research. 2023;26(3):320–30.

10. Reis LM, Maheshwari M, Capasso J, Atilla H, Dudakova L, Thompson S, et al. Axenfeld-Rieger syndrome: more than meets the eye. Journal of Medical Genetics. 2023 Apr 1;60(4):368–79.

11. Sheeladevi S, Lawrenson JG, Fielder AR, Suttle CM. Global prevalence of childhood cataract: a systematic review. Eye (Lond). 2016 Sep;30(9):1160–9.

12. Fan F, Luo Y, Wu J, Gao C, Liu X, Mei H, et al. The mutation spectrum in familial versus sporadic congenital cataract based on next-generation sequencing. BMC Ophthalmol. 2020 Sep 3;20:361.

13. Fernández-Alcalde C, Nieves-Moreno M, Noval S, Peralta JM, Montaño VEF, del Pozo Á, et al. Molecular and Genetic Mechanism of Non-Syndromic Congenital Cataracts. Mutation Screening in Spanish Families. Genes (Basel). 2021 Apr 16;12(4):580.

14. Sun W, Gu S, Zhang F, Xu M, Chang P, Zhao Y. Congenital cataracts affect the retinal visual cycle and mitochondrial function: A multi-omics study of GJA8 knockout rabbits. Journal of Proteomics. 2023 Sep 15;287:104972.

15. Carvalho DR, Medeiros JEG, Ribeiro DSM, Martins BJAF, Sobreira NLM. Additional features of Gillespie syndrome in two Brazilian siblings with a novel ITPR1 homozygous pathogenic variant. European Journal of Medical Genetics. 2018 Mar 1;61(3):134–8.

16. Hall HN, Williamson KA, FitzPatrick DR. The genetic architecture of aniridia and Gillespie syndrome. Hum Genet. 2019;138(8):881–98.

17. Keehan L, Jiang MM, Li X, Marom R, Dai H, Murdock D, et al. A novel de novo intronic variant in ITPR1 causes Gillespie syndrome. American Journal of Medical Genetics Part A. 2021;185(8):2315–24.

18. Lenhart PD, Lambert SR, Newman NJ, Biousse V, Atkinson DS, Traboulsi EI, et al. Intracranial Vascular Anomalies in Patients With Morning Glory Disk Anomaly. American Journal of Ophthalmology. 2006 Oct 1;142(4):644-650.e2.

19. Pavanello M, Fiaschi P, Accogli A, Severino M, Tortora D, Piatelli G, et al. A rare triad of morning glory disc anomaly, moyamoya vasculopathy, and transsphenoidal cephalocele: pathophysiological considerations and surgical management. Neurol Sci. 2021 Dec 1;42(12):5433–9.

20. Nguyen DT, Boddaert N, Bremond-Gignac D, Robert MP. Optic Nerve Abnormalities in Morning Glory Disc Anomaly: An MRI Study. Journal of Neuro-Ophthalmology. 2022 Jun;42(2):199.

21. Ozeki H, Shirai S, Nozaki M, Sakurai E, Mizuno S, Ashikari M, et al. Ocular and systemic features of Peters’ anomaly. Graefe’s Arch Clin Exp Ophthalmol. 2000 Oct 1;238(10):833–9.

22. Li Y, Zhang J, Dai Y, Fan Y, Xu J. Novel Mutations in COL6A3 That Associated With Peters’ Anomaly Caused Abnormal Intracellular Protein Retention and Decreased Cellular Resistance to Oxidative Stress. Frontiers in Cell and Developmental Biology [Internet]. 2020 [cited 2023 Aug 4];8. Available from: https://www.frontiersin.org/articles/10.3389/fcell.2020.531986

23. Stingl JV, Diederich S, Diel H, Schuster AK, Wagner FM, Chronopoulos P, et al. First Results from the Prospective German Registry for Childhood Glaucoma: Phenotype–Genotype Association. J Clin Med. 2021 Dec 21;11(1):16.

24. Chesneau B, Aubert-Mucca M, Fremont F, Pechmeja J, Soler V, Isidor B, et al. First evidence of SOX2 mutations in Peters’ anomaly: Lessons from molecular screening of 95 patients. Clinical Genetics. 2022;101(5–6):494–506.

25. Lim SH, Tran-Viet KN, Yanovitch TL, Freedman SF, Klemm T, Call W, et al. CYP1B1, MYOC, and LTBP2 Mutations in Primary Congenital Glaucoma Patients in the United States. American Journal of Ophthalmology. 2013 Mar 1;155(3):508-517.e5.

26. Yu-Wai-Man C, Arno G, Brookes J, Garcia-Feijoo J, Khaw PT, Moosajee M. Primary congenital glaucoma including next-generation sequencing-based approaches: clinical utility gene card. Eur J Hum Genet. 2018 Nov;26(11):1713–8.

27. Qiao Y, Chen Y, Tan C, Sun X, Chen X, Chen J. Screening and Functional Analysis of TEK Mutations in Chinese Children With Primary Congenital Glaucoma. Front Genet. 2021 Dec 10;12:764509.

28. Cronemberger S, Veloso AW, Lins P, Melo AC, da Silva AHG, de Figueiredo Barbosa L, et al. Outcomes of early versus delayed trabeculotomy for primary congenital glaucoma. Acta Ophthalmologica. 2023;101(5):514–20.

29. Nutt RJ, Dowlut MS, McLoone SF, McLoone E. Epidemiology and long-term outcomes of primary congenital glaucoma: a population-based study. Eye. 2023 Feb 7;1–6.
