# Supplementary material for: A bird’s eye view on the use of whole exome sequencing in rare congenital ophthalmic diseases
Source: J Hum Genet. 2024 Mar 8;69(6):271–82. doi: 10.1038/s10038-024-01237-6 (PMC11126393; doi:10.1038/s10038-024-01237-6)
Supplement: Supplementary file 2 — Supplementary Table 2 [file 10038_2024_1237_MOESM2_ESM.docx]

**Supplementary Table 2. Detailed information about the cohort and the results obtained by genetic testing.**

| ID | Disease | Inheritance | Method | Result of genetic testing | Gene | Variant | Genomic Position (hg19) |
| --- | --- | --- | --- | --- | --- | --- | --- |
| A1 | Aniridia | Sporadic | Sanger sequencing | Positive | *PAX6* | c.357C>G  p.Ser119Arg | chr11-31823109-G-C |
| A3 | Aniridia | Sporadic | Sanger sequencing | Positive | *PAX6* | c.607C>T  p.Arg203* | chr11-31816253-G-A |
| A6 | Aniridia | Sporadic | Sanger sequencing | Positive | *PAX6* | c.607C>T  p.Arg203* | chr11-31816253-G-A |
| A13 | Aniridia | Sporadic | Sanger sequencing | Positive | *PAX6* | c.916+1G>A  p.Arg203* | chr11-31815199-C-T |
| A18  A19  A29 | Aniridia | Familial | MLPA | Positive | *DCDC1, DNAJC24, ELP4, IMMP1L* | - | arr[GRCh37]11p13(30953737_31685847)x1 |
| A20 | Aniridia | Sporadic | Sanger sequencing | Positive | *PAX6* | c.357C>G  p.Ser119Arg | chr11-31823109-G-C |
| A23 | Aniridia | Sporadic | Sanger sequencing | Positive | *PAX6* | c.771G>A  p.Trp257* | chr11-31815345-C-T |
| A25 | Peters  Anomaly | Sporadic | WES | Positive | *PXDN* | c.970G>T  p.Gly324*  c.1357C>T  p.Gln453* | chr2-1677463-C-A  chr2-1668781-G-A |
| A28 | Aniridia | Sporadic | MLPA | Positive | *ARL14EP, DCDC1, DNAJC24, ELP4, FSHB, IMMP1L, MPPED2, PAX6, RCN1* | - | arr[GRCh37]11p13p14.1(30255684_31907122)x1 |
| A30 | Aniridia | Sporadic | WES | Positive | *PAX6* | c.357+1G>A  p.? | chr11-31823108-C-T |
| A35 | Aniridia | Sporadic | Sanger sequencing | Positive | *PAX6* | c.110_117del  p.Ala37Valfs*16 | chr11-31824276-CGGCCGGG- |
| A40  A85 | Aniridia | Sporadic | Sanger sequencing | Positive | *PAX6* | c.357+1G>A  p.? | chr11-31823108-C-T |
| A43  A55  A56  A57  A59 | Aniridia | Familial | MLPA | Positive | *DCDC1, DNAJC24, ELP4, IMMP1L* | - | arr[GRCh37]11p13(31030697_31755156)x1 |
| A44  A55 | Aniridia | Familial | WES | Inconclusive | *FOXC1* | c.1159G>C  p.Ala387Pro | chr6-1611604-G-C |
| A48  A49 | Aniridia | Familial | Sanger sequencing | Positive | *PAX6* | c.375_376dup  p.Val126Glufs*22 | chr11-31822386--CT |
| A51 | Aniridia | Sporadic | Sanger sequencing | Positive | *PAX6* | c.406C>T  p.Gln136* | chr11-31822356-G-A |
| A54 | Aniridia | Sporadic | Sanger sequencing | Positive | *PAX6* | c.244_245del  p.Glu82Serfs*9 | chr11-31823221TC-- |
| A61 | Aniridia | Sporadic | Sanger sequencing | Positive | *PAX6* | c.607C>T  p.Arg203* | chr11-31816253-G-A |
| A62 | Axenfeld-Rieger syndrome | Sporadic | WES | Negative |  |  |  |
| A63  A64 | Aniridia | Sporadic | MLPA | Positive | *DCDC1, DNAJC24, ELP4, IMMP1L* | - | arr[GRCh37]11p13(31212097_31751004)x1 |
| A65 | Aniridia | Sporadic | WES | Negative |  |  |  |
| A67 | Aniridia | Sporadic | Sanger sequencing | Positive | *PAX6* | c.3G>A  p.Met1Ile | chr11-31827957-C-T |
| A68 | Aniridia | Sporadic | WES | Positive | *PITX2* | c.416G>C  p.Trp139Ser | chr4-111539840-C-G |
| A69 | Aniridia | Familial | MLPA | Positive | *DCDC1, DNAJC24, ELP4, IMMP1L, PAX6, RCN1* | - | arr[GRCh37]11p13(31327164_31876477)x1 |
| A70 | Aniridia | Familial | Sanger sequencing | Positive | *PAX6* | c.781C>T  p.Arg261* | chr11-31815335-G-A |
| A71A  A71B | Aniridia | Familial | Sanger sequencing | Positive | *PAX6* | c.358-1G>C  p.? | chr11-31822405-C-G |
| A72 | Aniridia | Sporadic | Sanger sequencing | Positive | *PAX6* | c.180T>A  p.Tyr60* | chr11-31823286-A-T |
| A73 | WAGR | Sporadic | MLPA | Positive | *CCDC73, CSTF3, DCDC1, DEPDC7, DNAJC24, EIF3M, ELP4, IMMP1L, PAX6, PRRG4, QSER1, RCN1, TCP11L1, WT1* | - | arr[GRCh37]11p14.1p13(30638320_33182162)x1 |
| A74  A75 | Aniridia | Familial | Sanger sequencing | Positive | *PAX6* | c.607C>T  p.Arg203* | chr11-31816253-G-A |
| A81A  A81B  A81C  A81D | Aniridia | Familial | MLPA | Positive | *DCDC1, DNAJC24, ELP4, IMMP1L, PAX6* | - | arr[GRCh37]11p13(31329311_31828397)x1 |
| A82 | Aniridia | Sporadic | MLPA | Positive | *DCDC1, DNAJC24, ELP4, IMMP1L* | - | arr[GRCh37]11p14.1p13(30902664_31802443)x1 |
| A83 | Aniridia | Sporadic | Sanger sequencing | Positive | *PAX6* | c.520C>T  p.Gln174* | chr11-31822242-G-A |
| A84 | Aniridia | Sporadic | WES | Positive | *AMELX, ARHGAP6, ATXN3L, CLCN4, CLDN34, EGFL6, FAM9C, FRMPD4, GEMIN8, GLRA2, GPM6B, GPR143, HCCS, MID1, MSL3, OFD1, PRPS2, RAB9A, SHROOM2, TBL1X, TCEANC, TLR7, TLR8, TMSB4X, TRAPPC2, WWC3* | - | arr[GRCh37]Xp22.2(9651809_14770691)x1 |
| A86A  A86B  A86C  A86D  A86E  A86F | Aniridia | Familial | MLPA | Positive | *PAX6* | - | arr[GRCh37]11p13(31827945_31828010)x1 |
| A87 | Aniridia | Sporadic | Sanger sequencing | Positive | *PAX6* | c.718C>T  p.Arg240* | chr11-31815627-G-A |
| A88 | Aniridia | Sporadic | WES | Positive | *PAX6* | - | arr[GRCh37]11p13(31816347_31827834)x1 |
| A89 | Aniridia | Familial | Sanger sequencing | Positive | *PAX6* | c.718C>T  p.Arg240* | chr11-31815627-G-A |
| A91 | Aniridia | Sporadic | Sanger sequencing | Positive | *PAX6* | c.818dup  p.Asn273Lysfs*11 | chr11-31815298--T |
| A92 | Aniridia | Familial | Sanger sequencing | Positive | *PAX6* | c.1041_1053del  p.Ser349Hisfs*12 | chr11-31812388-GGTCTGGCTGGGG- |
| A94 | Aniridia | Sporadic | WES | Negative |  |  |  |
| A95 | Aniridia | Sporadic | Sanger sequencing | Positive | *PAX6* | c.357+1G>A  p.? | chr11-31823108-C-T |
| A96 | Aniridia | Familial | Sanger sequencing | Positive | *PAX6* | c.141+4A>G  p.? | chr11-31824248-T-C |
| A97 | Aniridia | Sporadic | WES | Positive | *MAB21L1* | c.155T>G  p.Phe52Cys | chr13-36050121-A-C |
| A98 | Aniridia | Sporadic | WES | Negative |  |  |  |
| A104 | Coloboma | Sporadic | WES | Negative |  |  |  |
| A106 | Coloboma | Sporadic | WES | Positive | *CAPN15* | c.2207G>A  p.Arg736Gln  c.2352C>A  p.Phe784Leu | chr16-601526-G-A + chr16-602057-C-A |
| A107 | Coloboma | Sporadic | WES | Negative |  |  |  |
| A108 | Aniridia | Sporadic | WES | Negative |  |  |  |
| A110 | Aniridia | Sporadic | Sanger sequencing | Positive | *PAX6* | c.764A>G  p.Gln255Arg | chr11-31815581-T-C |
| A112 | Coloboma | Sporadic | WES | Negative |  |  |  |
| A114 | Aniridia | Familial | MLPA | Inconclusive | *ELP4* | - | arr[GRCh37]11p13(31541660_31802443)x1 |
| A115 | Aniridia | Sporadic | WES | Positive | *PAX6* | c.158T>C  p.Val53Ala | chr11-31823308-A-G |
| A116 | Aniridia | Sporadic | Sanger sequencing | Positive | *PAX6* | c.551del  p.Gly184Glufs*23 | chr11-31816309-C- |
| A117  A241 | Aniridia | Sporadic | Sanger sequencing | Positive | *PAX6* | c.781C>T  p.Arg261* | chr11-31815335-G-A |
| A118 | Aniridia | Sporadic | Sanger sequencing | Positive | *PAX6* | c.183C>A  p.Tyr61* | chr11-31823283-G-T |
| A119 | Aniridia | Sporadic | Sanger sequencing | Positive | *PAX6* | c.949C>T  p.Arg317* | chr11-31815069-G-A |
| A121 | Morning Glory Anomaly | Sporadic | WES | Negative |  |  |  |
| A122 | Aniridia | Familial | MLPA | Positive | *PAX6, ELP4* | - | arr[GRCh37]11p13(31642266_31825698)x1 |
| A123 | WAGR | Sporadic | WES | Positive | *EIF3M, ELP4, PAX6, RCN1, WT1* | - | arr[GRCh37]11p13(31808455_32617592)x1 |
| A125 | Aniridia | Sporadic | Sanger sequencing | Positive | *PAX6* | c.607C>T  p.Arg203* | chr11-31816253-G-A |
| A130 | Aniridia | Sporadic | Sanger sequencing | Positive | *PAX6* | c.114_117del  p.Pro39Alafs*14 | chr11-31824276-CGGC- |
| A131 | Aniridia | Sporadic | Sanger sequencing | Positive | *ELP4* | c.1143+14176C>A | chr11-31685945-C-A |
| A133 | Coloboma | Sporadic | WES | Positive | *PXDN* | c.562C>T  p.Arg188*  c.3614A>G  p.Tyr1205Cys | chr2-1684133-G-A + chr2-1648519-T-C |
| A135 | Coloboma | Familial | WES | Negative |  |  |  |
| A136  A146 | Aniridia | Familial | Sanger sequencing | Positive | *PAX6* | c.357+1G>A  p.? | chr11-31823108-C-T |
| A137 | WAGR | Sporadic | MLPA | Positive | *ABTB2, ANO3, APIP, ARL14EP, BBOX1, BDNF, CAPRIN1, CAT, CCDC34, CCDC73, CD44, CD59, CSTF3, DCDC1, DEPDC7, DNAJC24, EHF, EIF3M, ELF5, ELP4, FBXO3, FIBIN, FJX1, FSHB,HIPK3, IMMP1L, KCNA4, KIAA1549L, KIF18A, LGR4, LIN7C, LMO2, METTL15, MPPED2, MUC15, NAT10, PAMR1, PAX6, PDHX, PRRG4, QSER1, RCN1, SLC1A2, SLC5A12, TCP11L1, TRIM44, WT1* | - | arr[GRCh37]11p14.2p13(26583085_35710586)x1 |
| A139 | Coloboma | Familial | WES | Negative |  |  |  |
| A140 | Aniridia | Familial | WES | Negative |  |  |  |
| A141 | Aniridia | Sporadic | WES | Positive | *ACTA2* | c.536G>A  p.Arg179His | chr10-90701066-C-T |
| A142 | Aniridia | Sporadic | Sanger sequencing | Positive | *PAX6* | c.765+1G>T  p.? | chr11-31815579-C-A |
| A143 | WAGR | Sporadic | MLPA | Positive | *ABTB2, ANO3, APIP, ARL14EP, BBOX1, BDNF, CAPRIN1, CAT, CCDC34, CCDC73, CD44, CD59, COMMD9, CSTF3, DCDC1, DEPDC7, DNAJC24, EHF, EIF3M, ELF5, ELP4, FBXO3, FIBIN, FJX1, FSHB, HIPK3, IFTAP, IMMP1L, KCNA4, KIAA1549L, KIF18A, LDLRAD3, LGR4, LIN7C, LMO2, METTL15, MPPED2, MUC15, NAT10, PAMR1, PAX6, PDHX, PRR5L, PRRG4, QSER1, RAG1, RAG2, RCN1, SLC1A2, SLC5A12, TCP11L1, TRAF6, TRIM44, WT1* | - | arr[GRCh37]11p14.3p12(25615512_39730342)x1 |
| A148 | Coloboma | Sporadic | WES | Negative |  |  |  |
| A151 | Aniridia | Sporadic | Sanger sequencing | Positive | *PAX6* | c.765+1G>C  p.? | chr11-31815579-C-G |
| A152  A156B  A156C | Aniridia | Familial | Sanger sequencing | Positive | *PAX6* | c.358-2A>G  p.? | chr11-31822406-T-C |
| A153 | Coloboma | Sporadic | WES | Positive | *TFAP2A* | c.1039T>C  p.Cys347Arg | chr6-10398698-A-G |
| A154  A257 | Aniridia | Sporadic | Sanger sequencing | Positive | *PAX6* | c.25_37del  p.Asn9Valfs*18 | chr11-31824356-CACCGAGCTGATT- |
| A162 | Aniridia | Sporadic | WES | Negative |  |  |  |
| A165 | Aniridia | Sporadic | WES | Positive | *PAX6* | c.-128-2del  p.? | chr11-31828475-T- |
| A167  A170 | Coloboma | Familial | WES | Positive | *FZD5* | c.236C>A  p.Ser79* | chr2-207768504-G-T |
| A168 | Aniridia | Sporadic | Sanger sequencing | Positive | *PAX6* | c.550G>T  p.Gly184* | chr11-31816310-C-A |
| A172 | Aniridia | Familial | MLPA | Positive | *DCDC1, DNAJC24, ELP4, IMMP1L* | - | arr[GRCh37]11p13(31176602_31714243)x1 |
| A173 | Coloboma | Sporadic | WES | Negative |  |  |  |
| A178  A179 | Aniridia | Familial | Sanger sequencing | Positive | *PAX6* | c.1068C>A  p.Cys356* | chr11-31812373-G-T |
| A180 | Aniridia | Sporadic | WES | Positive | *MAB21L1* | c.152G>A  p.Arg51Gln | chr13-36050124-C-T |
| A183 | Morning Glory anomaly | Sporadic | WES | Positive | *TUBA1A* | c.1169G>A  p.Arg390His | chr12-49578980-C-T |
| A189 | Coloboma | Sporadic | WES | Negative |  |  |  |
| A191 | Aniridia | Familial | Sanger sequencing | Positive | *PAX6* | c.1043_1056del  p.Pro348Leufs*18 | chr11-31812387-AGGTCTGGCTGGGG- |
| A192 | Aniridia | Familial | Sanger sequencing | Positive | *PAX6* | c.916+1G>A  p.? | chr11-31815199-C-T |
| A200 | Peters Anomaly | Sporadic | WES | Inconclusive | *SOX2* | c.611C>T  p.Ala204Val | chr3-181712971-C-T |
| A204  A225 | Aniridia | Familial | MLPA | Positive | *PAX6, ELP4* | - | arr[GRCh37]11p13(31540972_31813728)x1 |
| A205 | Aniridia | Sporadic | Sanger sequencing | Inconclusive | *PAX6* | c. 1267T>A  p.*423Lysext*14 | chr11-31789936-A-T |
| A208 | Coloboma | Sporadic | WES | Negative |  |  |  |
| A210 | Aniridia | Familial | WES | Positive | *ITPR1* | c.279+4_279+7delCGTA (homozygous)  p.? | chr3-4669561-ACGT- |
| A214 | Aniridia | Sporadic | WES | Positive | *PAX6* | c.1184-2A>G  p.? | chr11-31811569-T-C |
| A215  A216 | Rieger | Familial | Sanger sequencing | Positive | *PAX6* | c.682+2T>C  p.? | chr11-31816176-A-G |
| A217 | Peters Anomaly | Sporadic | WES | Negative |  |  |  |
| A219 | Coloboma | Sporadic | WES | Negative |  |  |  |
| A222 | Coloboma | Sporadic | WES | Negative |  |  |  |
| A223 | Aniridia | Sporadic | Sanger sequencing | Positive | *PAX6* | c.357+5G>A  p.? | chr11-31823104-C-T |
| A224A  A224B | Coloboma | Sporadic | WES | Negative |  |  |  |
| A226 | Aniridia | Sporadic | WES | Positive | *PAX6* | c.829C>T  p.Gln277* | chr11-31815287-G-A |
| A228 | Coloboma | Sporadic | WES | Negative |  |  |  |
| A230 | Coloboma | Sporadic | WES | Negative |  |  |  |
| A231 | Axenfeld-Rieger syndrome | Sporadic | WES | Negative |  |  |  |
| A232 | Aniridia | Sporadic | Sanger sequencing | Positive | *PAX6* | c.1268A>T  p.*423Leuext*14 | chr11-31811483-T-A |
| A233 | Aniridia | Familial | WES | Negative |  |  |  |
| A234 | Peters Anomaly | Sporadic | WES | Negative |  |  |  |
| A237 | Coloboma | Sporadic | WES | Positive | *PTPN11*  *ACAD8, B3GAT1, GLB1L2, GLB1L3, IGSF9B, JAM3, NCAPD3, SPATA19, THYN1, VPS26B* | c.1508G>A  p.Gly503Glu  - | chr12-112926888-G-A  arr[GRCh37]11q25(133711934_135006515)x1 |
| A240 | Aniridia | Sporadic | Sanger sequencing | Positive | *PAX6* | c.766-2A>T  p.? | chr11-31815352-T-A |
| A242 | Aniridia | Sporadic | WES | Positive | *PAX6* | c.1A>G  p.Met1Val | chr11-31827959-T-C |
| A243 | Morning Glory Anomaly | Familial | WES | Negative |  |  |  |
| A244 | Aniridia | Sporadic | WES | Positive | *CYP1B1* | c.352C>T  p.Pro118Ser  c.1064_1076delGAGTGCAGGCAGA  p.Arg355Hisfs*69 | chr2-38302180-G-A  chr2-38298421-TCTGCCTGCACTC-- |
| A245 | Aniridia | Sporadic | Sanger sequencing | Positive | *PAX6* | c.120C>A  p.Cys40* | chr11-31824273-G-T |
| A246 | Coloboma | Sporadic | WES | Negative |  |  |  |
| A247 | Aniridia | Familial | Sanger sequencing | Positive | *PAX6* | c.183delC  p.Tyr61* | chr11-31823283-G- |
| A253 | Aniridia | Sporadic | WES | Positive | *ITPR1* | c.7660G>A  p.Gly2554Arg | chr3-4856205-G-A |
| A256 | Aniridia | Sporadic | WES | Inconclusive | *DYNLRB2, MAF, WWOX* | - | arr[GRCh37]16q23.1q23.2(79030057_80574915)x1 |
| A258 | Aniridia | Sporadic | WES | Positive | *ITPR1* | c.7666G>A  p.Gly2556Arg | chr3-4856211-G-A |
| A259  A270  A271 | Aniridia | Familial | Sanger sequencing | Positive | *PAX6* | c.859_862dup  p.Ser288Asnfs*4 | chr11-31815254--TGAT |
| A260  A262  A269 | Aniridia | Familial | Sanger sequencing | Positive | *PAX6* | c.1265del  p.Gln422Argfs*103 | chr11-31811486-T- |
| A263 | Aniridia | Sporadic | Sanger sequencing | Positive | *PAX6* | c.111_121delinsT  p.Arg38Thrfs*13 | chr11-31824272-CGCACGGCCGG-A |
| A264 | Aniridia | Sporadic | WES | Positive | *PAX6* | c.109del  p.Ala37Profs*17 | chr11-31824284-C- |
| A265 | Aniridia | Sporadic | WES | Positive | *PAX6* | c.433_443del  p.Lys145Valfs*51 | chr11-31822319-ATCCTTAGTTT- |
| A266 | Aniridia | Sporadic | WES | Positive | *PAX6* | c.1183G>A  p.Gly395Arg | chr11-31812258-C-T |
| A273 | Aniridia | Familial | WES | Inconclusive | *PAX6* | c. 1267T>A  p.*423Lysext*14 | chr11-31789936-A-T |
| A274 | Aniridia | Sporadic | Sanger sequencing | Positive | *PAX6* | c.763C>T  p.Gln255* | chr11-31815582-G-A |
| A276 | Aniridia | Sporadic | WES | Positive | PAX6 | c.749_763del  p.Pro250_Ile254del | chr11-31815582-GTATTCTTGCTTCAG- |
| A277 | Axenfeld-Rieger syndrome | Sporadic | WES | Negative |  |  |  |

MLPA: Multiplex ligation-dependent probe amplification; Positive: assessment of a pathogenic or likely pathogenic variant; Inconclusive: assessment of a variant of unknown significance; Negative: no variant was assessed in relation to the limitations of the methodology.
