# Supplementary material for: A bird’s eye view on the use of whole exome sequencing in rare congenital ophthalmic diseases
Source: J Hum Genet. 2024 Mar 8;69(6):271–82. doi: 10.1038/s10038-024-01237-6 (PMC11126393; doi:10.1038/s10038-024-01237-6)
Supplement: Supplementary file 3 — Supplementary Table 3 [file 10038_2024_1237_MOESM3_ESM.docx]

**Supplementary Table 3. Quality values related to samples analyzed by WES.**

| ID | Mean Coverage | Region covered >20X | Gene | Variant | Frequency (gnomAD NFE) | Allelic Balance | Variant Coverage |
| --- | --- | --- | --- | --- | --- | --- | --- |
| A25 | 122X | 96.6% | PXDN | c.970G>T  p.Gly324*  c.1357C>T  p.Gln453* | 0.00000885  - | VAF: 0.47  VAF: 0.55 | 148X  121X |
| A30 | 82X | 97.4% | PAX6 | c.357+1G>A  p.? | - | VAF: 0.4 | 109X |
| A44 | 115X | 97.6% | *FOXC1* | c.1159G>C  p.Ala387Pro | - | VAF: 0.32 | 88X |
| A45 | 103X | 97.2% | *FOXC1* | c.1159G>C  p.Ala387Pro | - | VAF: 0.59 | 59X |
| A62 | 83X | 97.6% | - | - | - | - | - |
| A65 | 64X | 94.3% | - | - | - | - | - |
| A68 | 111X | 97.7% | PITX2 | c.416G>C  p.Trp139Ser | - | VAF: 0.57 | 57X |
| A84 | 92X | 97.6% | AMELX, ARHGAP6, ATXN3L, CLCN4, CLDN34, EGFL6, FAM9C, FRMPD4, GEMIN8, GLRA2, GPM6B, GPR143, HCCS, MID1, MSL3, OFD1, PRPS2, RAB9A, SHROOM2, TBL1X, TCEANC, TLR7, TLR8, TMSB4X, TRAPPC2, WWC3 | - | - | CN: 0.92 | - |
| A88 | 110X | 99.6% | PAX6 | - | - | CN: 1 | - |
| A94 | 120X | 94.6% | - | - | - | - | - |
| A97 | 82X | 97.3% | *MAB21L1* | c.155T>G¥  p.Phe52Cys | - | VAF: 0.47 | 92X |
| A98 | 94X | 97.6% | - | - | - | - | - |
| A104 | 112X | 97.3% | - | - | - | - | - |
| A106 | 83X | 97.5% | CAPN15 | c.2207G>A  p.Arg736Gln  c.2352C>A  p.Phe784Leu | -  - | VAF: 0.61  VAF: 0.42 | 57X  38X |
| A107 | 96X | 97.4% | - | - | - | - | - |
| A108 | 100X | 95.4% | - | - | - | - | - |
| A112 | 85X | 97.4% | - | - | - | - | - |
| A115 | 116X | 90.6% | PAX6 | c.158T>C  p.Val53Ala | - | VAF: 0.51 | 74X |
| A121 | 126X | 97.8% | - | - | - | - | - |
| A123 | 91X | 97.5% | EIF3M, ELP4, PAX6, RCN1, WT1 | - | - | CN: 0.97 | - |
| A133 | 84X | 97.4% | PXDN | c.562C>T  p.Arg188*  c.3614A>G  p.Tyr1205Cys | -  - | VAF:0.47  VAF: 0.44 | 45X  63X |
| A135 | 79X | 97.3% | - | - | - | - | - |
| A139 | 92X | 97.6% | - | - | - | - | - |
| A140 | 94X | 97.4% | - | - | - | - | - |
| A141 | 79X | 97.4% | ACTA2 | c.536G>A  p.Arg179His | - | VAF: 0.5 | 117X |
| A148 | 86X | 97.4% | - | - | - | - | - |
| A153 | 90X | 97.5% | TFAP2A | c.1039T>C  p.Cys347Arg | - | VAF: 0.53 | 32X |
| A162 | 99X | 97.7% | - | - | - | - | - |
| A165 | 91X | 97.6% | PAX6 | c.-128-2del  p.? | - | VAF: 0.34 | 53X |
| A167 | 108X | 97.6% | FZD5 | c.236C>A  p.Ser79* | - | VAF:0.51 | 140X |
| A170 | 117X | 97.7% | FZD5 | c.236C>A  p.Ser79* | - | VAF:0.48 | 92X |
| A173 | 87X | 97.2% | - | - | - | - | - |
| A180 | 80X | 97.4% | MAB21L1 | c.152G>A  p.Arg51Gln | - | VAF:0.41 | 102X |
| A183 | 114X | 97.8% | TUBA1A | c.1169G>A  p.Arg390His | - | VAF: 0.5 | 178X |
| A189 | 93X | 97.5% | - | - | - | - | - |
| A200 | 86X | 97.4% | *SOX2* | c.611C>T  p.Ala204Val | - | VAF: 0.41 | 61X |
| A208 | 93X | 97.5% | - | - | - | - | - |
| A210 | 93X | 97.6% | ITPR1 | c.279+4_279+7delCGTA  p.? | - | VAF: 1 | 43X |
| A214 | 89X | 97.6% | *PAX6* | c.1184-2A>G  p.? | - | VAF: 0.58 | 33X |
| A217 | 84X | 97.5% | - | - | - | - | - |
| A219 | 78X | 96.9% | - | - | - | - | - |
| A222 | 79X | 97.3% | - | - | - | - | - |
| A224A | 86X | 97.5% | - | - | - | - | - |
| A224B | 85X | 97.5% | - | - | - | - | - |
| A226 | 96X | 97.6% | *PAX6* | c.829C>T  p.Gln277* | - | VAF: 0.5 | 121X |
| A228 | 91X | 97.4% | - | - | - | - | - |
| A230 | 94X | 97.5% | - | - | - | - | - |
| A231 | 107 | 97.7% | - | - | - | - | - |
| A233 | 75X | 97.5% | - | - | - | - | - |
| A234 | 91X | 97.6% | - | - | - | - | - |
| A237 | 82X | 97.3% | PTPN11  ACAD8, B3GAT1, GLB1L2, GLB1L3, IGSF9B, JAM3, NCAPD3, SPATA19, THYN1, VPS26B | c.1508G>A  p.Gly503Glu  - | -  - | VAF: 0.39  CN: 1.01 | 84X  - |
| A242 | 89X | 97.6% | *PAX6* | c.1A>G  p.Met1Val | - | VAF: 0.52 | 60X |
| A243 | 94X | 97.5% | - | - | - | - | - |
| A244 | 95X | 97.7% | CYP1B1 | c.352C>T  p.Pro118Ser  c.1064_1076delGAGTGCAGGCAGA  p.Arg355Hisfs*69 | 0.0000109  0.000371 | VAF: 0.46  VAF: 0.31 | 76X  42X |
| A246 | 75X | 97.4% | - | - | - | - | - |
| A253 | 128X | 97.7% | ITPR1 | c.7660G>A  p.Gly2554Arg | - | VAF: 0.51 | 80X |
| A256 | 91X | 99.75% | DYNLRB2, MAF, WWOX | - | - | CN: 1 | - |
| A258 | 86X | 97.6% | ITPR1 | c.7666G>A  p.Gly2556Arg | - | VAF: 0.4 | 40X |
| A264 | 73X | 97.2% | *PAX6* | c.109del  p.Ala37Profs*17 | - | VAF: 0.5 | 83X |
| A265 | 82X | 97.6% | *PAX6* | c.433_443del  p.Lys145Valfs*51 | - | VAF: 0.5 | 62X |
| A266 | 67X | 97.8% | *PAX6* | c.1183G>A  p.Gly395Arg | - | VAF: 0.5 | 54X |
| A273 | 142X | 99.7% | *PAX6* | c. 1267T>A  p.*423Lysext*14 | RF | VAF:0.22 | 59X |
| A276 | 66X | 97.3% | PAX6 | c.749_763del  p.Pro250_Ile254del | - | VAF: 0.31 | 45X |
| A277 | 93X | 97.3% | - | - | - | - | - |

# VAF: Variant allele frequency; CN: Copy Number; RF: Failed random forest filtering thresholds in gnomAD; ¥ published in Hall et al. 2022 (22).
